# Molecular signatures of multiple myeloma progression through single cell RNA-Seq

**DOI:** 10.1038/s41408-018-0160-x

**Published:** 2019-01-03

**Authors:** Jin Sung Jang, Ying Li, Amit Kumar Mitra, Lintao Bi, Alexej Abyzov, Andre J. van Wijnen, Linda B. Baughn, Brian Van Ness, Vincent Rajkumar, Shaji Kumar, Jin Jen

**Affiliations:** 1 Genome Analysis Core, Medical Genome Facility, Center for Individualized Medicine, Mayo Clinic, Rochester, MN USA; 2 Division of Bioinformatics and Biostatistics, Department of Health Science Research, Mayo Clinic, Rochester, MN USA; 3 Department of Drug Discovery and Development, Harrison School of Pharmacy, Auburn University, Auburn, AL USA; 4 Division of Hematology, Department of Internal Medicine, Mayo Clinic, Rochester, MN USA; 5 Department of Orthopedic Surgery, Mayo Clinic, Rochester, MN USA; 6 Department of Laboratory Medicine and Pathology, Mayo Clinic, Rochester, MN USA; 7 Department of Genetics, Cell Biology & Development, University of Minnesota, Minneapolis, MN USA

## Abstract

We used single cell RNA-Seq to examine molecular heterogeneity in multiple myeloma (MM) in 597 CD138 positive cells from bone marrow aspirates of 15 patients at different stages of disease progression. 790 genes were selected by coefficient of variation (CV) method and organized cells into four groups (L1–L4) using unsupervised clustering. Plasma cells from each patient clustered into at least two groups based on gene expression signature. The L1 group contained cells from all MGUS patients having the lowest expression of genes involved in the oxidative phosphorylation, Myc targets, and mTORC1 signaling pathways (*p* < 1.2 × 10^−14^). In contrast, the expression level of these pathway genes increased progressively and were the highest in L4 group containing only cells from MM patients with *t*(4;14) translocations. A 44 genes signature of consistently overexpressed genes among the four groups was associated with poorer overall survival in MM patients (APEX trial, *p* < 0.0001; HR, 1.83; 95% CI, 1.33–2.52), particularly those treated with bortezomib (*p* *<* 0.0001; HR, 2.00; 95% CI, 1.39–2.89). Our study, using single cell RNA-Seq, identified the most significantly affected molecular pathways during MM progression and provided a novel signature predictive of patient prognosis and treatment stratification.

## Introduction

Multiple myeloma (MM) is a malignant hematological disorder characterized by the accumulation of terminally differentiated antibody-secreting plasma cells with clonal genetic/cytogenetic abnormalities that home to the bone marrow^1–3^. Clinically, monoclonal gammopathy of undetermined significance (MGUS) is a pre-neoplasic condition preceding MM. Extensive immunophenotypic and differential gene expression analyses have shown that MGUS and MM can also be distinguished from normal plasma cells but not from each other^4^.

Fluorescence in situ hybridization (FISH) studies of neoplastic plasma cells demonstrate trisomies of multiple odd numbered chromosomes in ~40% of MM cases, while the majority of the remaining cases have a translocation involving the immunoglobulin heavy chain (IgH) gene at chromosome 14q32^1–3^. Secondary cytogenetic abnormalities can also occur during the disease progression, including gains of 1q, deletions of 17p (resulting in *TP53* loss) and 13q, as well as mutations and secondary translocations involving *MYC*^1,2^. Both primary and secondary cytogenetic abnormalities, as well as specific molecular alterations are known to influence disease progression, response to therapy, and prognosis^1^. There is strong evidence of genetic heterogeneity based on the diverse pattern of molecular changes including clonal evolution and differential clonal response, which impact prognostic stratification, therapeutic approaches, and disease response to treatment^2,5,6^. High throughput single cell RNA-Seq (scRNA-Seq) technology offers an opportunity for an unbiased gene expression profiling of plasma cells obtained from each patient to understand the pathogenesis of MM progression that can better guide patient prognosis and selection for appropriate clinical interventions^7^.

In this study, we performed scRNA-Seq using 597 cells derived from 15 patients at different stages of MM including MGUS, smoldering multiple myeloma (SMM), newly diagnosed MM (NDMM), and relapsed and/or refractory MM (RRMM). At the resolution of single cells, we identified gene expression signatures and molecular pathways relating to disease progression that are present within each patient and affect overall survival (OS).

## Materials and methods

### MM samples, plasma cell selection, and cell capture for scRNA-Seq

**Fig. 1:**
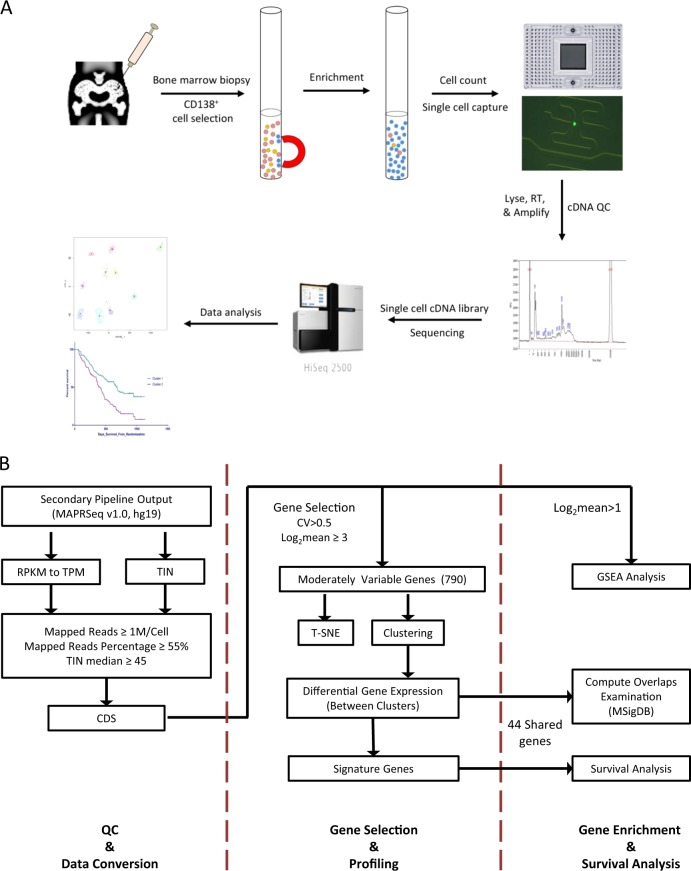
Workflow of scRNA-Seq in MM. **a** Schematic illustration of scRNA-Seq from bone marrow aspirate. **b** Bioinformatics analysis pipeline which includes three components: QC and data conversion, gene selection and profiling, and clustering and survival analyses

Bone marrow aspirates were collected from 15 patients (Table 1) after informed consent and subjected to ACK lysis and mononuclear cell isolation. Plasma cells were separated by positive selection using CD138-coated magnetic beads (MACS; Miltenyi Biotec, CA) in a RoboSep system (STEMCELL Technology, Canada). CD138-positive cells were examined using Vi-CELL XR (Beckman Coulter, CA) to determine cell number, viability, and average size. A microfluidic mRNA-Seq chip (Fluidigm, CA) was used for capturing cells from each sample at a concentration of 500 cells/μl and run in the Fluidigm C1 system to generate double-stranded cDNA using SMARTer Ultra Low RNA kit for Illumina (Takara, CA). All samples were assessed for cell capture in a C1 chip by direct observation under a microscope and for cDNA quality using Fragment Analyzer (HS Large Fragment kit, Advanced Analytical Technologies, IA). In total, 701 single cells that generated cDNA fragments > 1000 bp on average were included in the subsequent sequencing analysis. While the exact protocol was used, we observed a significant variation on the total number of cells isolated from each bone marrow biopsy and the number of cells captured by the Fluidigm C1 chip. This is in part due to individual sample variations, as well as possible difference in disease stage, since the total number of captured plasma cells was overall lower in MGUS cases compared to those at SMM or MM stages (Table 1). Overall, the number of analyzable cells from each patient reflected the total number of CD138-positive cells available, as well as the quality of the bone marrow biopsy. Figure 1a outlines the major steps of this study. Aliquots of the same bone biopsy were also retained and analyzed by FISH as a part of the routine clinical diagnosis and extracted from patients’ pathology record following institutional approved IRBs.

**Table 1:** Patient characteristics and distribution of cells based on 790 genes

Sample IDs	Total number of cells sequenced	Total number of cells passing QC and analyzed	L 1	L 2	L3	L4	Cytogenetic abnormality information
IgM MGUS1	26	24 (92%)	15 (63%)	6 (25%)	3 (13%)		Not tested
IgM MGUS3	33	17 (52%)	16 (94%)	1 (6%)			Not tested
MGUS5	19	7 (37%)	4 (57%)	3 (43%)			Normal
SMM0	84	77 (92%)		10 (13%)		67 (87%)	*t*(4;14), gain 1q21, del 13q
SMM2	40	16 (40%)	14 (88%)	2 (12%)			*t*(14;20), monosomy 13
SMM3	40	39 (98%)		4 (10%)	34 (90%)		Trisomy 7, 9, 11 and 15
SMM4	51	44 (86%)		18 (41%)	26 (59%)		Trisomy 3, 7, 9, 11, 14, & 15
NDMM3	65	59 (91%)	3 (5%)	27 (46%)	29 (49%)		Trisomy 3, 7, & 11, trisomy/tetrasomy 9 & 15
NDMM5	41	32 (78%)	1 (3%)	30 (94%)	1 (3%)		Trisomy 7, 9, 11, & 14, trisomy/tetrasomy 3 &15, del 13q
NDMM6	48	47 (98%)	3 (6%)	41 (88%)	3 (6%)		Trisomy 3, 9, 11, & 15
NDMM7	59	54 (92%)	30 (56%)	24 (44%)			*t*(11;14)
NDMM8	63	60 (95%)		26 (43%)	34 (57%)		Trisomy 3, 8, 9, & 14, trisomy/tetrasomy 7, tetrasomy 11, gain 1q21
RRMM1	48	46 (96%)		1 (2%)		45 (98%)	*t*(4;14), monosomy 13, del 17p
RRMM2	50	42 (84%)	1 (2%)	12 (28%)	28 (67%)	1 (2%)	*t*(4;14), trisomy 11 &15, monosomy 9 &13
RRMM4	34	33 (97%)	1 (3%)	32 (97%)			*t*(11;14) and tetraploid
Total	701	597 (85%)	89 (15%)	237 (40%)	158 (26%)	113 (19%)

### scRNA-Seq library construction and sequencing

For RNA-Seq library construction, single cell cDNAs (250 pg) were used to construct indexed libraries using Nextera XT DNA Sample Preparation kit (Illumina, CA). Libraries were quantified by Bioanalyzer (High Sensitivity DNA analysis kit, Agilent, CA) and Qubit (dsDNA BR Assay kits, Life Technologies, CA). Single cell libraries obtained from each patient were pooled at up to 48 cells per lane and sequenced using the 101 bases paired-end protocol on Illumina HiSeq 2500 Rapid Run. FASTQ formatted raw files from each sample were mapped to the hg19.

### scRNA-Seq data QC and analysis

We used MAPRSeq (v1.0)^8^ to analyze RNA-Seq data as outlined in Fig. 1b with TopHat2 for reads alignment to the hg19 and FeatureCounts for gene expression. During quality control (QC), we consider cells of low quality if the total number of reads/cell < 1,000,000, percentage of mapped reads <55%, and if the median of non-zero gene transcript integrity number (TIN) < 45^9^. Of the 701 single cell libraries sequenced, 597 passed QC and were included in the downstream analysis. We observed that some genes have reads mostly aligned to the 3′UTR with few reads aligned to the coding DNA sequence (CDS) regions. To reduce bias in gene expression based on 3′UTR alignments, we used only expression in CDS for subsequent analysis^10^. Transcripts per kilobase million (TPM) was used as the measure for gene expression. Raw sequence data and processed data sets from this study have been submitted to Gene Expression Omnibus (GEO; http://www.ncbi.nlm.nih.gov/geo/) under accession number GSE118900).

**Fig. 2:**
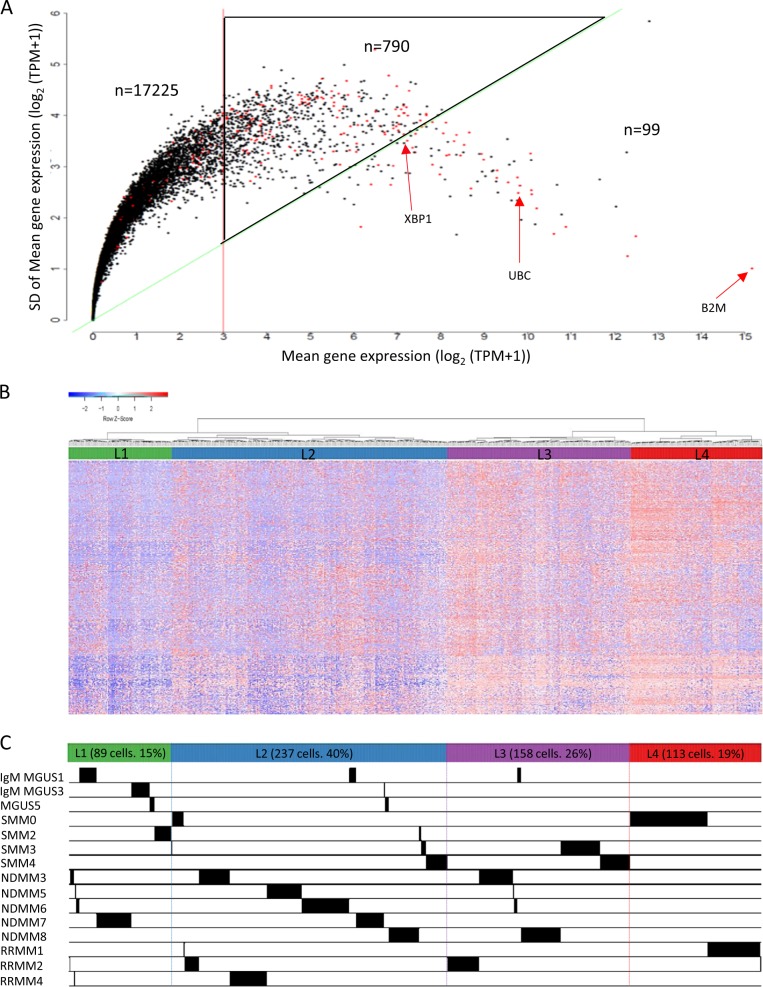
Identification of signature genes and hierarchical clustering analysis (HCA). **a** Selection of genes (*n* = 790, inside black triangle) with coefficient of variation (CV) ≥ 0.5 (slope of the triangle) and a mean average gene expression value log_2_ (TPM + 1) ≥ 3 (vertical line of the triangle). Red dots: housekeeping genes (HG). **b** Unsupervised two-dimensional HC analysis using the 790 genes. Four main branches were formed (L1–L4) by HCA. **c** Distribution plot of a total of 597 cells from 15 patients. Each black line indicates each cell

For gene selection and molecular signature analyses, we first performed Seurat *t*-distribution stochastic neighborhood embedding (*t*-SNE, V1.2) and clustering analysis^11^ using genes expressed in more than two cells with log_2_mean > 1 and standard deviation (*y*) > 1 (Supplemental Figure S1). Secondly, we employed a coefficient of variation (CV)^12^ approach to select for highly variable genes with CV ≥ 0.5 and log_2_(TPM + 1) ≥ 3 as the cutoff. Of the 790 genes in this group (Supplemental Table S1), 14.8% (*n* = 117) were housekeeping genes (HGs) as categorized by Hsiao et al.^13^. In contrast, 40% (*n* = 39) were HGs among the 99 genes with the high expression levels (log_2_(TPM + 1) > 3) and low CV values CV ≤ 0.5). Using this approach, we excluded HGs uniformly expressed in a majority of the cells while reducing the stochastic noise associated with low copy transcripts to capture the most significant differential gene expression signatures among the individual MM cells (Fig. 2a).

Unsupervised hierarchical clustering was performed based on the expression levels of the 790 selected genes using the “1—Pearson correlation” distance and Ward.D2 linkage approach. ANOVA analysis was performed among the major groups of cells (log_2_ scale) and the significantly differentially expressed genes were selected using ANOVA *p*-value < 0.05 and two-fold change (FC) (Supplement Figure S2). To further understand the biological characteristics of the data set, Compute Overlaps Examination of Molecular Signatures Database v5.2 (MsigDB) was carried out using 311 common genes and 44 signature genes that are significantly up-regulated (FC ≥ 2, *p* < 0.05) within each comparison using L1 as reference. Hallmarks gene set and C5 GO gene sets in MsigDB (v5.2) were used to identify the most significant pathways within the data set.

### Survival analysis using publically available datasets

We extracted microarray gene expression (GEP) data from APEX trial^14,15^ on 44 with consistently increased expression among L1–L4 groups. For genes with multiple probe sets, mean intensities were computed. GEP data was then mean-centered and scaled prior to analysis. Unsupervised *K*-means clustering was performed using Hartigan and Wong’s algorithm^16^ based on the GEP signature. Kaplan–Meier (KM) survival curves were generated for the clusters by computing OS over time. The KM curves were compared and *p*-values were generated using Mantel–Cox log-rank test.

## Results

### Patient population and clinical status

Our scRNA-Seq analysis included CD138-positive cells isolated from bone aspirates of patients with MGUS (*n* = 3), SMM (*n* = 4), NDMM = 5, and RRMM (*n* = 3). A total of 701 cells were collected using the Fluidigm C1 nanofluidics platform and 597 cells passed QC and included in the analyses. An average of 16 cells from three MGUS patients were included in the analysis (range, 7–24 cells/patient) while an average of 46 cells/patient were analyzed for those at either SMM or MM stages of the disease (range, 16–77 cells/patient and 40–50 cells/MM subgroup). Consistent with the clinical status of the disease, MGUS cases collectively had fewer analyzable CD138-positive plasma cells compared those in later stages. A summary of the clinical information and FISH results obtained as a part of routine clinical practice for all patients are shown in Table 1.

### Gene selection and molecular classification of MM based on scRNA-Seq

We first used *t*-SNE analysis to assess cell-to-cell variability after sample QC of scRNAseq data and observed that most plasma cells clustered primarily by individual patients reflecting the highly clonal nature of MM, except MGUS group (Supplemental Figure S1). We next used CV method to select 790 genes with moderately high expression values of log_2_(TPM + 1) ≥ 3 and ≥ 0.5 fold variation (CV) in gene expression across all 597 cells from 15 patients (Fig. 2a). By unsupervised hierarchical clustering (Fig. 2b), cells self-organized into four main clusters (L1–L4) each composed of cells from patients at different stages of MM diagnosis (Fig. 2b). Cells in the L1 group were characterized by low level expression in genes involved in the oxidative phosphorylation, Myc targets, and mTORC1 signaling when compared to the other groups (*p* < 1.2 × 10^−14^, Supplemental Table S2).

As shown in Table 1, most CD138-positive cells from the three MGUS patients clustered into the L1 group (63%) with other cells in the L2 or L3 groups (29% and 9%, respectively**)**. When compared with cytogenetic abnormalities identified by FISH, all cells from patients with either *t*(14;20) or *t*(11;14) translocation clustered into L1 and L2 groups (SMM2, NDMM7, and RRMM4). In contrast, a majority of cells from patients with trisomy features (SMM3 and 4, NDMM3, 5, 7, and 8) belonged to the L2 and L3 groups while cells with *t*(4;14) translocations appeared to contribute exclusively to the L4 cluster (SMM0, RRMM1 and 2). Sample RRMM2, with both *t*(4;14) and trisomies of 11 and 15, had cells that cluster across all four clusters. In total, 89 cells formed L1 cluster (15%) with a gene expression signature representing low-risk MM having a high representation of cells from MGUS and cytogenetically favorable patients. Gene expression profiles from 237 cells in the L2 group (40%) and 113 cells in L3 (26%) more closely represented cytogenetically complex MM based on their association with cases having trisomies or tetrasomies. Lastly, gene expression signature from cells in the L4 group (113 cells, 19%) appeared to reflect the highest risk MM given it is exclusively of cells obtained from the patients having the *t*(4;14) translocation (Fig. 2c and Table 1).

### Altered protein homeostasis genes among subgroups

**Fig. 3:**
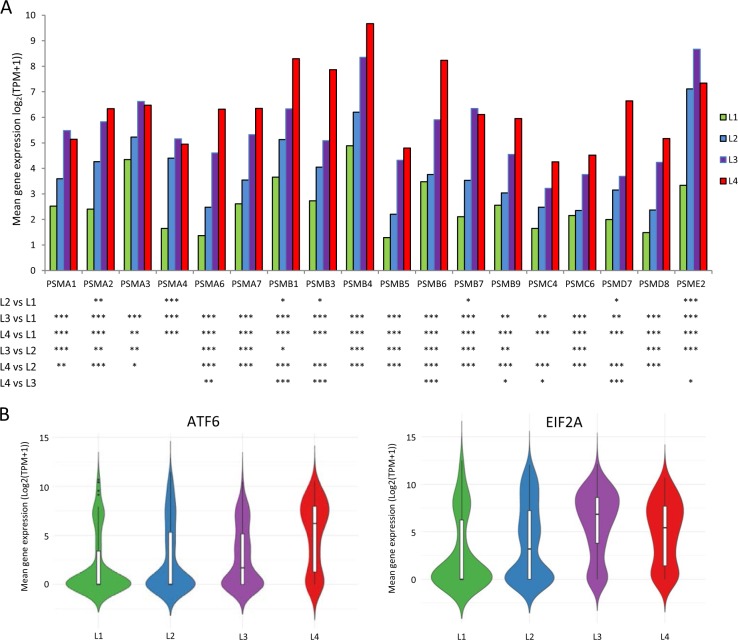
Expression of protein homeostasis genes among clustering cell groups. **a** Relative expression for 18 proteasome subunits genes in L1–L4 groups. *p*-values and fold changes by ANOVA are shown in Supplemental Table S3. **b** Relative expression of *ATF6* and *EIF2A* genes within each single cell group. Vertical axis is the log-transformed mean expression values and width indicates frequency of cells at the indicated expression level. *p < 0.05; **p < 0.01; ***p < 0.001

Since it is well established that MM patients often respond well to proteasome inhibitor therapy, we examined the expression status of all 18 genes encoding the proteasome subunits in the 790 selected genes. Each of these genes was differentially over expressed when compared between the groups (Fig. 3a). At *p* < 0.05, seven proteasome genes (*PSMA2, PSMA4, PSMB1, PSMB3, PSMB7, PSMD7*, and *PSME2*) were significantly highly expressed in L2 (FC, 2.2–13.7) compared to L1, while all 18 genes were significantly up-regulated in both L3 and L4 groups (FC, 3.0–40.4) when compared to cells in L1 group (Supplemental Table S3). Similarly, when comparing the L3 and L4 groups to L2, a majority of the proteasome genes were highly expressed in the later groups (FC, 2.6–22.3). Furthermore, eight proteasome genes (*PSMA6, PSMB1, PSMB3, PSMB6*, *PSMB9, PSMC4, PSMD7*, and *PSME2*) were expressed at remarkably higher levels (FC, 3.0–7.2) in L4 compared to L3. Additionally, critical genes involved in the unfolded protein response pathway (UPR, also known as ER stress response) were also significantly up-regulated. One of those, *XBP1* is stably expressed at high levels in a majority of the cells analyzed (CV ≤ 0.5 and Fig. 2a). *ATF6*, a transcription factor that activates target genes for UPR, was predominantly expressed at a high level in the L4 group while *EIF2A* (Eukaryotic translation Initiation Factor 2A) was significantly highly expressed in both the L3 and L4 groups (Fig. 3b).

### Molecular pathways involved in MM progression

**Fig. 4:**
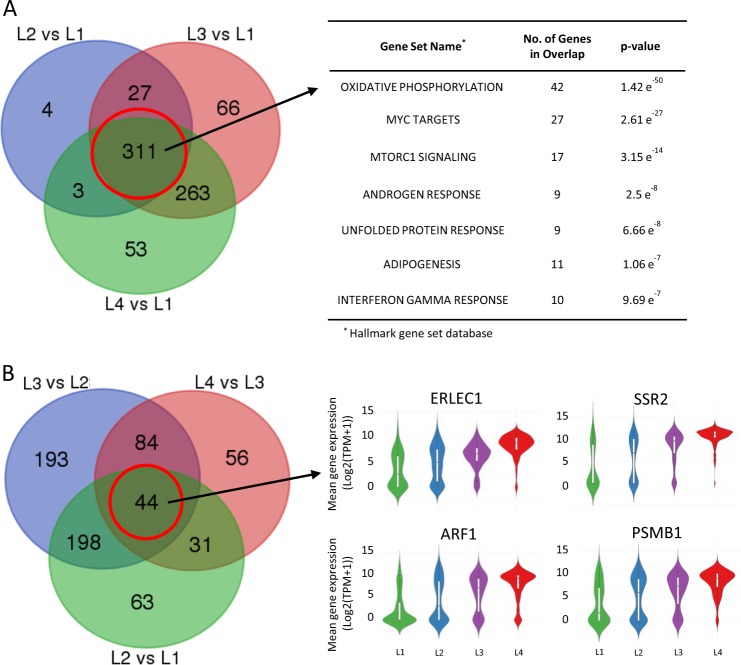
Differential expression genes and associated pathways with MM Progression. **a** Most significantly up-regulated (FC ≥ 2, *p* < 0.05) and shared 311 genes when comparing each cell groups to L1. **b** Identification of 44 genes with most consistently altered in expression levels (FC ≥ 2, *p* *<* 0.05) between the adjacent groups and sample violin plots for 4 of 44 shared genes (red circle)

Comparing cells in the L1 group to each of the higher cell clustering groups (L2–L4), we obtained a total of 311 common genes most significantly up-regulated from L1 to L4 groups (*p* *<* 0.05, FC ≥ 2, Fig. 4a and Supplemental Table S4). Compute Overlaps Examination of MSigDB showed that gene sets shared among these groups were associated with cell metabolism and protein homeostasis, such as oxidative phosphorylation, Myc-targeted genes, mTORC1 signaling, and UPR (Fig. 4a). When considering genes significantly altered in expression levels (FC ≥ 2, *p* *<* 0.05) between the adjacent groups, out of 311 common genes, we identified a 44 signature genes with consistently increased expression level among the groups (Fig. 4b). Using GO term analysis, we found that 26/44 (59%) were related genes with UPR pathway, function of endoplasmic reticulum and mitochondria that highlighting their role in MM (Supplemental Table S5).

### Clinical implications of genes associated with MM progression

**Fig. 5:**
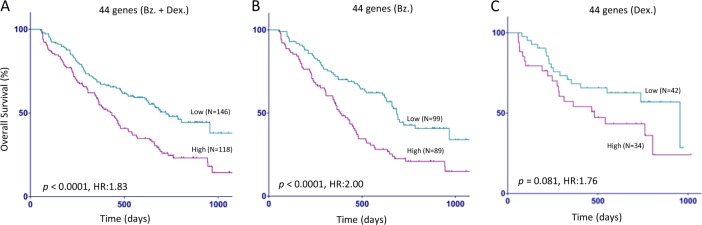
Survival analysis using 44 signature gene sets. Microarray gene expression data from APEX (**a**–**c**) was used and Kaplan–Meier (KM) survival curve are shown based on the high and low expression status of the signature genes. *p*-values were generated using Mantel–Cox log-rank test. Bz. Bortezomib; Dex. Dexamethasone, HR hazard ratio, *Y*-axis percentage of survival, *X*-axis days of survival from randomization

We examine the clinical association of the 44 genes most consistently associated with MM progression from pair-wise comparisons between the four groups (L1 vs. L2, L2 vs. L3, and L3 vs. L4) to examine whether the expression patterns of these genes correlate with OS in MM patients. Using the APEX trial data set and when dichotomized as high and low expression groups, the 44 gene expression signature was able to distinguish OS in all patients (*p* < 0.0001; hazard ratio (HR), 1.831; 95% CI, 1.33–2.522). Strikingly, this survival significance was primarily observed in the bortezomib treatment group (*p* *<* 0.0001; HR, 2.001; 95% CI, 1.387–2.888) but not in patients treated with dexamethasone (*p* < 0.0812; HR, 1.763, 95% CI, 0.9133–3.403; Fig. 5).

## Discussion

Single cell RNA-Seq is a powerful tool to identify unique cell types and unmask the cellular heterogeneity in the tumor microenvironment^17,18^. However, scRNA-Seq data can be inherently noisy due to pre-amplification of single cell RNA and the stochastic nature of RNA transcription^19,20^. Data analysis to identify underlying biological variations with confidence is further confounded by the large gene expression variations within a cell, and the lower coverage per transcriptome in general when the total reads are distributed over a large number of individual cells rather than a single mixed cell population. In the context of MM, most transcriptome profiling studies to date have focused on CD138-selected plasma cells from bone marrow aspirates. Gene expression changes from pooled cells represent an average expression and could mask gene expression signatures by subpopulations of cells with high expression^18,21–23^. In addition, the highly monoclonal nature of the MM disease posts a significant challenge in assessing intercellular heterogeneity even at the resolution of single cells.

To overcome these technical challenges, we utilized several different analytical approaches for gene expression analysis in single cells. By *t*-SNE^11^ we observed that most cells clustered exclusively by individual patients reflecting the clonal genetic changes unique to each patient. We used the CV approach^12^ to focus on robustly expressed genes with a variation of CV ≥ 0.5 (*n* = 790), thus reducing technical and biological noises for subsequent unsupervised clustering analyses. Using this strategy, we observed that cells from all 15 patients re-clustered into four sub-populations (L1–L4) based on the gene expression obtained by scRNA-Seq (Table 1). Cells derived from each patient’s bone biopsy dispersed across different clusters reflecting the heterogeneous nature of plasma cells in MM (Fig. 2b).

A novel observation from our study is that while a highly clonal disease, plasma cells derived from each of the 15 patients have diverse molecular signatures that corresponded to different stages of disease and reflected cytogenetic features observed in MM at different risk levels. Although a relatively small study involving a few hundred cells, most cells in the L1 group displayed a relatively the lowest level of activations for genes involved in oxidative phosphorylation and proteasome homeostasis (Figs. 3a and 4a). L2 and L3 contained a majority of the cells from patients with trisomies and cytogenetically complexed MM (Table 1). L4 group is exclusive of cells from patients with *t*(4;14). It is worth noting that proteasome genes were some of the most prominently expressed in L3 and L4 groups and their expression was successively increased compared to cells in the L1 and L2 groups (Fig. 3). These results are consistent with the favorable responses observed in some MM patients treated with proteasome inhibitor.

Recently, there is increasing evidence of interplay between UPR and the mammalian target of rapamycin (mTOR) kinase signaling pathway^24–26^. Both mTOR and UPR pathways control many cellular processes including apoptosis, translation, energy metabolism, and inflammation^24–27^. mTOR regulates cell growth, survival, proliferation, and metabolism. In this study, we observed that mTORC1 pathway genes were not only significantly enriched and elevated in the high-risk cell cluster, but also likely serve as a distinct feature with activation of UPR, glycolysis, and protein secretion pathways between the high risk of trisomies MM (L3) and *t*(4;14) RRMM (L4) groups (Table 1 and Supplemental Table S2). These observations are supported by several reports on the crosstalk between UPR and mTORC1 pathway in the cells contributing to malignancy cells^14,16–18^ and provide the mechanistic rationale to mTOR inhibitors as a useful regimen to potentially improve treatment efficacy for MM and RRMM in combination with proteasome inhibitors^24,26–28^.

The clinical relevance of our single cell analysis is highlighted by the strong association of the 44 gene signature with OS from MM in independent clinical studies (Fig. 5). Significantly, 26 of the 44 genes consistently overexpressed among the four groups (Fig. 4b) were related to protein homeostasis and energy metabolism function and likely contributed to treatment response and OS in patients treated with proteasome inhibitors (Supplemental Table S5).

In conclusion, we used scRNA-Seq to examine gene expression signature of individual plasma cells (*n* = 597) from 15 patients at different stages of MM progression. We showed that different fractions of CD138-positive cells from each of the 15 patients clustered into four main groups corresponding to increasing risk levels in MM. Compared to the minimal risk cluster which contained most of the plasma cells derived from MGUS patients, increased expression of genes involved in protein homeostasis in MM cells in the later groups (L2–L4) is associated with progression and reduced survival of MM. Although our study is limited to only 15 patients and a total of 597 cells, comprehensive bioinformatics analyses of this high-resolution molecular dataset enabled us to derive at a robust molecular signature in MM progression at the resolution of single cells that reflect different risk levels across all samples. Our findings will require validation in a larger patient cohort, use of sequentially obtained plasma cells during the course of MGUS to MM progression, as well as improvements in analytical capabilities by analyzing many more cells per patients and the inclusion of unique molecular indexes (UMIs) during the cDNA synthesis to validate whether combination regimens including proteasome inhibitors and mTOR inhibitors could improve MM treat and overcome drug resistance in MM and RRMM. It will also be highly informative to determine whether the fraction of plasma cells within different risk clusters change over time in individual MM patients and how they affect disease progression, treatment response, and patient outcome.

## Electronic supplementary material

Supplemental Table S1

Supplemental Table S2

Supplemental Table S3

Supplemental Table S4

Supplemental Table S5

Supplemental Figure S1

Supplemental Figure S2

Supplemental Figure Legends
