# Supplementary material for: Molecular signatures of multiple myeloma progression through single cell RNA-Seq
Source: Blood Cancer J. 2019 Jan 3;9(1):2. doi: 10.1038/s41408-018-0160-x (PMC6318319; doi:10.1038/s41408-018-0160-x)
Supplement: Supplementary file 1 — Supplemental Table S1 [file 41408_2018_160_MOESM1_ESM.pdf]

Supplemental Table S1. 790 genes list with moderately highly variable genes (log2(TPM+1).

GeneID	L1.Average	L2.Average	L3.Average	L4.Average
ABCF1	2.478	2.772	3.478	3.139
ACADVL	2.324	2.322	3.653	4.947
ACTG1	2.890	4.422	5.684	5.436
ACTR3	1.916	2.779	4.176	4.138
AFTPH	2.235	2.482	3.515	4.147
AIG1	0.786	2.082	3.668	6.508
AKAP13	2.775	3.686	4.691	3.124
AKAP2	1.458	2.896	3.967	3.629
AKR1A1	2.145	2.878	4.225	5.583
ALDOA	3.776	4.267	6.482	9.045
ALG5	2.581	2.636	4.309	4.660
ANAPC11	1.674	2.302	3.723	5.045
ANAPC16	2.809	4.113	5.389	7.353
ANAPC5	3.566	3.920	5.986	5.264
ANKHD1	3.976	4.421	5.005	4.486
ANKHD1-EIF4EBP3	3.937	4.356	4.918	4.383
ANKRD10	4.655	5.481	6.542	5.912
ANKRD12	4.729	4.659	5.322	5.084
ANKRD28	4.666	5.141	5.338	5.804
ANP32B	1.879	3.563	5.354	3.218
ANXA2	1.794	4.004	5.628	7.420
ANXA5	3.090	4.157	4.794	7.339
ANXA7	2.262	2.453	2.947	4.349
APRT	1.362	3.279	4.063	5.374
ARF1	2.353	4.303	5.870	7.796
ARF4	3.865	4.957	6.153	8.815
ARFGAP3	2.589	2.785	3.395	6.009
ARHGDIB	2.407	3.622	4.176	6.169
ARID4B	2.680	4.055	3.951	4.071
ARID5B	2.194	2.985	3.291	2.898
ARL6IP5	3.514	3.078	4.140	5.741
ARPC2	5.043	6.605	7.759	7.978
ARPC3	2.345	4.154	6.040	7.073
ASH1L	2.694	2.538	2.867	4.543
ATF6	1.791	2.505	2.660	5.057
ATG3	1.489	3.104	4.680	4.213
ATM	2.828	2.824	3.989	3.118
ATOX1	1.767	2.983	4.903	6.117
ATP11B	2.412	3.228	3.947	2.830
ATP5A1	3.322	5.402	7.292	6.794
ATP5B	3.472	5.723	7.343	7.906
ATP5C1	1.509	2.539	4.983	5.109
ATP5F1	1.859	2.780	4.145	5.565
ATP5G1	1.965	3.971	5.593	8.351
ATP5G2	3.462	6.215	8.321	7.888
ATP5G3	2.585	3.819	6.622	7.787
ATP5H	2.552	4.111	5.813	5.911
ATP5J	3.670	4.972	6.983	8.249
ATP5J2	2.864	5.189	7.869	7.501
ATP5J2-PTCD1	1.420	2.831	4.546	4.294
ATP5O	2.670	4.557	7.088	7.564
ATP6V0B	2.449	2.667	4.490	7.135
ATP6V0E1	3.508	5.453	7.490	8.224
ATP6V1F	1.212	2.140	4.964	5.097
ATP6V1G1	3.580	4.856	7.533	7.001
ATPIF1	2.579	3.410	5.921	7.179
AUP1	2.218	3.319	4.792	5.696
BANF1	0.288	2.880	5.298	6.416
BAZ2B	2.573	2.875	3.885	3.740
BBX	2.317	3.621	3.744	3.110
BCLAF1	5.903	5.554	5.997	6.980
BET1	1.939	2.485	3.768	4.551
BHLHE41	2.589	2.913	3.760	4.414
BLNK	4.537	3.268	4.432	5.070

BOD1L	2.597	3.004	3.365	3.244
BPTF	2.900	2.972	3.649	3.724
BRD2	3.439	4.017	5.027	5.785
BRK1	1.776	2.766	4.723	4.545
BRP44L	2.134	2.410	4.084	6.429
BSCL2	0.957	2.131	5.266	6.402
BSG	1.714	2.646	3.821	3.957
BST2	1.775	3.871	5.618	3.643
BTAF1	2.359	3.256	4.273	3.473
BTF3	3.548	5.660	7.426	7.538
BTG1	5.256	4.198	4.771	4.157
BTG2	4.898	6.491	7.907	9.151
BUB3	1.912	2.968	5.213	4.444
BUD31	1.242	2.618	4.244	3.748
C11orf31	0.777	2.853	4.506	3.975
C11orf58	3.859	6.257	7.747	6.771
C12orf57	2.536	3.728	5.420	7.851
C14orf166	1.657	2.658	4.347	4.669
C14orf2	2.051	4.503	6.923	6.896
C15orf63	1.668	3.493	6.063	4.629
C19orf10	2.418	3.748	6.159	6.927
C19orf42	1.398	2.685	4.705	4.340
C19orf53	1.858	3.426	5.530	4.826
C19orf70	0.806	2.533	4.994	4.554
C19orf79	3.614	6.183	8.769	8.340
C1orf63	2.891	3.866	3.721	3.267
C22orf32	1.677	2.257	4.299	4.301
C2orf28	2.428	3.600	6.156	8.626
C4orf3	2.637	3.122	4.110	5.343
C6orf48	1.145	2.459	5.413	2.598
C6orf62	4.219	5.621	6.697	7.871
C7orf23	2.608	4.562	5.882	5.169
C7orf59	1.771	2.925	4.971	5.638
C8orf59	1.843	3.242	5.001	5.124
CADPS2	2.026	3.183	3.675	3.253
CALM1	5.811	6.023	7.492	8.579
CALM2	4.116	6.359	7.875	9.088
CALR	3.705	3.268	5.136	6.400
CALU	2.080	3.399	4.889	5.628
CANX	2.894	5.140	6.769	6.334
CAPZA1	3.400	3.734	4.754	6.404
CASP3	2.529	3.452	4.181	3.384
CAST	2.269	4.394	5.394	4.886
CBWD1	1.759	3.479	5.153	5.066
CBWD2	1.873	3.239	4.633	4.974
CCDC144A	1.437	3.338	3.705	3.198
CCNDBP1	2.307	3.939	5.888	4.375
CCNH	2.753	3.391	3.427	3.963
CCNI	2.208	3.683	4.769	5.068
CCT3	1.441	2.451	3.521	5.333
CCT7	1.544	2.656	3.568	3.541
CCT8	1.639	3.507	5.144	3.551
CD164	1.703	2.486	3.080	5.320
CD38	4.595	5.024	6.340	7.008
CD44	3.117	4.048	2.970	3.062
CD46	3.327	4.517	5.670	7.270
CD48	1.944	4.288	6.543	7.787
CD53	3.282	5.142	7.114	8.696
CD55	3.537	2.707	4.418	4.495
CD59	2.903	3.671	6.306	7.197
CD63	5.877	6.635	8.302	10.081
CDC26	2.109	2.736	4.167	3.282
CDK14	3.133	2.023	2.942	4.852
CDK2AP2	1.611	3.044	4.135	7.674
CDK5RAP3	3.866	3.424	4.455	4.216
CENPC1	2.736	3.106	3.739	3.064
CEP350	2.452	2.924	3.555	4.043
CFL1	1.889	2.781	4.404	5.532

CFLAR	2.782	4.009	4.095	7.943
CHCHD2	2.491	4.163	6.455	7.783
CHD1	2.484	3.088	3.474	2.684
CHD2	3.867	4.244	4.856	3.620
CHID1	1.349	2.398	4.582	5.330
CHMP2A	1.979	3.404	5.477	5.311
CHURC1	1.618	2.797	4.648	4.545
CIR1	2.175	2.852	3.629	3.862
CIRBP	1.602	3.215	4.728	2.935
CKLF	1.569	2.996	3.567	5.308
CKLF-CMTM1	1.462	2.692	3.200	4.876
CLIC1	2.604	4.672	6.487	8.777
CLINT1	4.276	5.110	5.924	6.006
CLPTM1L	1.597	3.172	4.244	6.013
CNBP	3.025	4.760	5.755	6.622
CNPY2	2.813	3.889	5.745	7.139
COMMD3	6.486	7.479	8.095	8.361
COMMD6	2.735	2.806	3.939	4.761
COPA	2.741	2.423	3.382	4.885
COPB1	3.501	4.618	6.099	5.698
COPB2	1.591	3.045	3.603	4.255
COPS6	1.664	2.660	4.595	4.371
COX16	1.826	2.938	4.341	4.528
COX17	2.837	5.410	6.576	7.564
COX20	1.244	2.631	4.354	4.350
COX5A	1.529	3.770	6.241	4.736
COX5B	2.407	5.253	7.684	7.154
COX6A1	4.860	7.015	8.662	9.787
COX6B1	3.510	6.367	9.064	8.906
COX6C	3.755	6.174	8.625	9.171
COX7A2	4.203	6.280	8.281	9.047
COX8A	1.491	3.779	6.698	7.242
CPEB4	2.569	2.841	4.795	2.755
CPNE1	3.905	4.191	5.727	6.783
CRBN	1.697	3.612	3.533	2.624
CREB3L2	1.860	4.182	5.687	5.310
CSDE1	2.387	3.859	4.961	5.605
CSNK1A1	1.618	2.558	3.574	3.945
CSNK2B	1.622	2.897	4.212	5.133
CSPP1	2.193	2.572	3.114	3.894
CTSS	3.537	4.228	6.705	7.811
CUTA	3.728	5.625	7.592	7.670
CWC15	1.537	2.704	4.172	3.156
CXCR4	3.882	3.012	4.509	4.861
CYBA	3.309	4.251	5.542	6.559
CYCS	3.006	4.308	5.630	7.867
CYTIP	5.769	5.577	6.774	5.472
DAD1	5.056	5.929	7.912	10.225
DALRD3	2.331	2.806	3.739	2.603
DAP3	1.257	3.069	4.109	6.285
DBI	2.155	3.658	5.703	7.359
DDIT3	4.325	3.019	2.024	4.954
DDOST	3.436	3.402	6.031	8.023
DDX17	3.188	4.116	4.831	4.825
DDX18	2.099	3.475	4.165	4.119
DDX21	2.584	3.615	4.723	4.053
DDX24	4.218	5.029	4.787	5.323
DDX6	3.653	4.865	6.768	5.060
DENND4A	2.156	2.796	4.185	3.047
DERL1	2.859	3.573	4.564	5.141
DERL2	1.654	2.108	3.367	6.076
DHRS7	2.374	3.673	5.344	6.203
DHX36	2.364	3.804	3.869	2.948
DMTF1	1.933	3.271	3.887	3.454
DNAJA1	3.901	5.098	5.177	4.858
DNAJB11	1.762	4.252	4.484	6.552
DNAJB14	4.083	4.943	6.334	6.015
DNAJB9	2.333	3.226	4.841	7.141

DNAJC1	3.960	5.060	5.673	5.527
DNAJC10	2.049	3.456	5.305	7.099
DNAJC15	2.046	2.801	5.387	2.489
DNAJC3	3.304	4.878	4.859	7.670
DNAJC8	1.968	2.662	4.125	4.863
DOCK8	2.010	2.965	4.133	2.955
DPM3	1.561	2.257	3.235	7.573
DST	1.480	2.967	3.075	4.389
DSTN	1.905	2.912	4.709	6.578
DUSP1	4.235	4.334	4.731	6.820
DUSP22	3.080	2.473	4.543	3.147
DYNLL1	2.576	5.401	6.823	7.864
DYNLRB1	0.975	2.624	3.688	5.911
EAF2	3.777	6.247	6.248	8.186
EAPP	2.480	2.766	4.021	4.076
ECH1	2.240	3.145	5.100	5.933
EDF1	4.551	5.692	8.005	8.189
EEF2	2.466	2.929	4.941	4.117
EID1	1.887	3.640	5.041	4.829
EIF2A	2.585	3.817	5.905	4.863
EIF2AK3	3.598	4.723	5.588	3.989
EIF2AK4	2.564	3.112	3.936	3.030
EIF2S2	3.445	4.508	5.582	6.761
EIF3A	4.363	5.124	6.589	5.740
EIF3D	1.469	2.537	4.363	3.268
EIF3E	2.022	5.066	7.150	7.148
EIF3F	1.795	2.473	5.386	4.440
EIF3G	1.978	3.084	5.695	3.752
EIF3H	1.933	2.963	5.123	4.075
EIF3I	2.828	2.073	4.364	4.766
EIF3J	1.662	4.103	5.067	3.217
EIF3K	3.382	5.776	7.802	6.442
EIF3L	2.072	3.360	5.667	4.415
EIF3M	2.506	3.880	5.905	3.839
EIF4A1	2.434	2.528	4.144	3.561
EIF4A2	5.771	6.310	7.765	5.465
EIF4G2	3.677	4.473	5.594	5.822
EIF5	4.081	4.621	5.108	6.692
EIF5B	3.374	4.309	5.128	5.232
ELF1	3.209	3.329	4.009	2.776
ELL2	3.358	5.174	5.746	4.997
ELP2	2.836	3.585	4.382	2.943
EMP3	2.103	2.664	5.015	7.178
ENO1	1.106	2.253	3.538	5.451
ENY2	2.137	2.357	4.126	4.434
EPRS	3.726	5.265	6.010	6.627
ERGIC2	3.090	3.177	4.907	7.457
ERGIC3	3.734	3.846	6.850	9.871
ERLEC1	3.326	4.644	6.122	8.366
ERP29	2.243	2.714	3.933	4.217
ERP44	1.508	2.487	4.269	5.088
ETF1	2.435	3.179	5.031	2.710
EVI2A	2.265	3.566	4.660	6.043
EVI2B	4.032	5.925	7.398	7.505
EWSR1	3.371	3.128	3.564	4.063
EZR	5.578	4.932	6.130	6.480
FAM133B	3.333	4.462	5.093	3.586
FAM214A	2.367	3.966	5.599	3.484
FAM46C	3.530	4.633	5.160	6.423
FAM96A	2.354	2.518	4.556	4.835
FCHSD2	2.346	3.978	6.037	3.474
FCRL5	2.969	4.090	5.476	6.765
FCRLA	1.733	2.567	2.333	5.965
FIS1	1.568	2.627	4.385	4.444
FNBP4	2.045	3.221	4.172	3.296
FNDC3A	4.903	4.986	4.647	5.419
FNDC3B	4.410	4.959	6.044	5.089
FOS	7.526	7.254	7.604	9.903

FUT8	2.621	1.923	3.434	4.577
FXYD5	4.076	6.353	8.310	8.690
GABARAP	4.799	5.048	7.214	8.407
GAPDH	4.460	6.178	8.319	9.737
GARS	2.385	3.950	5.020	4.741
GHITM	1.531	2.517	3.332	5.465
GLCCI1	3.692	5.351	7.490	6.006
GLG1	2.537	2.644	3.543	5.429
GLO1	1.652	2.645	3.821	4.982
GLRX	1.151	3.641	5.785	6.752
GLS	3.644	3.647	3.952	3.146
GMFG	2.725	3.031	5.002	3.524
GNG5	2.150	3.576	4.895	6.777
GNG7	1.861	3.681	5.719	5.543
GNL3	3.112	4.901	5.880	6.509
GOLGB1	4.170	4.522	5.531	5.355
GPBP1	2.873	4.672	5.592	3.761
GPCPD1	3.724	2.597	4.395	3.482
GPRC5D	0.533	3.047	4.191	8.441
GPX4	2.737	3.178	5.650	4.843
GSTK1	1.831	2.619	4.745	4.754
GSTP1	7.271	5.486	6.949	8.868
GTF2A2	0.662	2.612	4.396	4.362
GUK1	1.505	2.400	4.372	6.559
H2AFZ	2.056	2.999	4.647	7.172
HAX1	2.848	3.560	5.210	8.783
HBP1	2.723	2.900	4.024	3.238
HCLS1	2.438	3.437	3.884	3.114
HIGD2A	1.708	3.808	6.393	5.159
HIST1H1C	2.875	4.822	6.504	5.125
HIST1H2AC	1.168	3.064	4.137	2.947
HIST1H2BC	1.977	3.300	3.964	4.904
HIST1H2BK	1.705	3.639	4.748	4.732
HIST1H4C	4.948	6.011	7.499	8.252
HLA-A	4.375	5.916	7.008	6.269
HLA-DOB	2.019	2.399	4.227	6.638
HLA-E	4.788	6.538	7.624	7.330
HLA-F	2.031	3.491	3.648	5.723
HLA-G	2.182	3.414	3.856	3.981
HM13	3.250	3.930	4.964	6.887
HMGN2	3.263	3.665	4.476	5.499
HMGN3	4.841	6.383	7.356	7.109
HNRNPA1	4.513	6.394	8.278	6.734
HNRNPA1L2	3.238	4.655	6.251	5.011
HNRNPA2B1	5.817	6.520	7.819	7.799
HNRNPA3	2.311	3.087	3.625	4.372
HNRNPC	3.278	5.071	6.388	7.557
HNRNPD	2.840	2.985	3.687	4.123
HNRNPH1	4.737	4.188	5.173	4.128
HNRNPM	1.945	2.787	4.331	2.355
HNRNPR	1.846	2.838	3.857	4.193
HNRNPU	3.091	4.667	5.951	5.781
HNRPDL	3.345	4.439	5.968	6.796
HP1BP3	2.173	2.873	3.470	3.876
HSH2D	3.032	2.458	4.183	3.766
HSPA9	2.003	3.637	4.601	3.947
HSPD1	2.532	3.518	5.108	4.008
HSPE1	1.822	3.534	5.672	4.944
HSPE1-MOB4	2.096	2.948	4.683	4.297
IARS	2.178	3.341	5.429	4.399
ICAM2	2.351	2.792	3.693	4.898
ICAM3	1.290	3.066	5.109	3.413
IER3IP1	1.146	2.535	4.362	5.986
IFI16	2.683	3.682	4.797	4.878
IFITM1	3.119	7.957	9.051	2.381
IFITM2	0.891	4.409	4.696	3.354
IFITM3	1.877	5.942	6.346	2.424
IFNAR1	2.539	3.665	4.795	6.213

IFRD1	3.328	4.548	5.067	4.876
IFT20	3.018	2.844	4.882	4.074
IGF1	3.043	2.706	3.978	3.014
IK	2.483	3.739	5.091	3.971
IL6ST	4.205	4.637	4.948	4.712
ILF2	1.847	3.066	3.797	6.026
ING3	2.243	2.795	4.810	4.081
IQCB1	2.371	2.800	3.450	3.557
IRF4	2.984	4.010	4.550	3.554
ISCU	2.037	3.061	4.703	6.753
ISG20	4.713	7.036	8.162	8.151
ITGA4	2.427	3.951	4.694	4.655
ITGA8	2.794	2.819	3.713	3.523
ITM2B	4.252	5.784	6.010	5.296
JMJD1C	3.639	3.777	4.836	4.588
JTB	1.739	2.995	4.745	6.484
JUN	5.269	4.487	4.501	4.594
KDELR1	2.313	2.284	5.426	5.553
KDELR2	1.601	2.755	4.442	5.563
KDM2A	2.322	2.830	4.257	3.491
KIAA0368	2.148	3.227	3.378	3.727
KLF6	6.568	5.269	6.302	8.015
KNTC1	3.247	2.456	4.482	3.549
KTN1	2.965	3.155	3.653	4.277
LARS	1.451	3.271	3.861	4.033
LDHA	1.599	2.715	4.844	5.879
LDHB	2.663	4.113	7.469	6.571
LGALS1	3.244	3.663	4.229	8.864
LMAN1	4.013	5.243	5.784	7.814
LMAN2	2.733	3.848	5.847	8.059
LRMP	3.809	2.906	4.421	4.319
LRRC16A	3.278	3.561	4.443	3.533
LSM3	2.539	3.022	4.345	5.256
LSM5	1.121	2.778	4.624	4.758
LSMD1	1.825	2.705	4.632	5.501
LUC7L2	2.830	3.544	4.986	4.139
LUC7L3	3.093	4.830	5.245	5.036
MAN1A2	3.428	3.748	5.044	6.778
MAN2A1	2.143	3.814	4.450	3.257
MANF	1.994	2.790	4.022	9.394
MAPK1IP1L	2.996	3.017	4.532	3.659
6-Mar	3.771	3.646	5.279	3.933
MARS	2.137	2.677	3.877	3.951
MATR3	3.255	4.884	5.264	4.874
MBD4	1.699	3.387	3.523	3.182
MCFD2	2.061	2.775	3.770	5.222
MDH1	1.747	2.637	3.953	7.677
MDM4	2.516	2.941	3.434	4.810
MED31	2.364	2.598	2.811	4.590
MEF2C	3.717	4.008	5.161	3.895
MEI1	3.913	4.640	5.649	6.933
MESDC2	2.793	4.969	7.042	7.496
MGEA5	3.015	4.169	4.923	3.883
MGST3	1.462	2.839	4.200	7.306
MIA3	2.122	2.311	3.220	4.588
MIER1	2.021	3.076	3.550	4.055
MLL3	2.964	3.983	4.800	3.761
MLLT3	2.494	4.825	4.449	2.953
MMADHC	2.293	2.499	4.259	4.763
MORF4L1	4.682	5.608	7.121	6.133
MPHOSPH8	3.542	3.943	4.407	3.139
MRPL20	1.643	2.719	4.360	4.884
MRPL32	1.418	2.319	4.361	4.746
MRPL33	1.673	3.298	4.102	4.974
MRPL51	2.500	3.014	5.118	7.207
MRPL53	1.475	2.799	4.410	4.547
MRPS21	2.456	4.255	6.943	6.049
MRPS24	2.193	3.753	7.464	7.725

MRPS31	2.710	2.791	4.518	2.125
MTIF3	2.611	3.309	4.174	2.741
MTRNR2L2	4.289	5.819	7.575	5.818
MYCBP2	3.001	3.648	4.310	3.542
MYL12A	2.460	5.097	5.555	8.692
MYL12B	2.508	3.552	4.814	6.191
MYO5A	2.203	2.408	4.011	2.915
N4BP2L2	4.640	4.440	4.885	3.949
NAA38	1.889	2.826	4.245	3.706
NAMPT	4.077	4.775	5.344	6.185
NAP1L1	4.644	5.549	6.267	6.866
NAP1L4	2.134	2.420	4.315	3.626
NARS	1.480	2.943	3.948	4.117
NCL	3.686	4.882	5.945	5.921
NCOA3	3.350	3.329	4.694	3.813
NDUFA11	2.265	4.613	6.791	6.622
NDUFA12	1.238	2.349	4.683	5.615
NDUFA13	5.019	6.777	8.313	8.694
NDUFA2	1.911	3.552	6.247	5.521
NDUFA4	3.781	6.941	9.523	9.632
NDUFA6	2.030	3.412	5.548	6.107
NDUFA7	1.561	2.561	4.860	4.979
NDUFAB1	1.305	2.089	4.309	5.567
NDUFAF3	1.390	3.378	4.518	6.030
NDUFB1	1.947	4.696	6.038	6.023
NDUFB10	1.236	2.833	3.401	4.622
NDUFB2	2.726	4.716	7.362	6.494
NDUFB3	2.199	3.536	5.758	6.969
NDUFB4	3.904	6.525	7.698	7.794
NDUFB6	0.887	2.886	5.200	4.853
NDUFB7	1.957	4.074	6.191	6.104
NDUFB8	2.130	4.337	7.027	7.490
NDUFB9	2.604	4.013	6.541	6.282
NDUFC1	3.291	4.647	6.601	6.452
NDUFC2	2.197	4.859	6.701	6.556
NDUFC2-KCTD14	1.920	4.521	6.335	6.194
NDUFS5	2.632	3.650	7.174	6.686
NDUFS6	1.651	3.440	4.903	3.706
NDUFS8	1.569	3.088	4.910	4.786
NDUFV2	3.441	4.889	6.585	6.931
NEDD8	2.242	4.378	6.724	7.434
NEDD8-MDP1	2.296	4.061	5.900	6.900
NEMF	2.839	3.762	3.645	4.117
NFKBIA	5.449	6.896	7.144	8.811
NGLY1	2.637	3.848	5.106	4.936
NHP2	1.311	3.195	5.018	5.846
NHP2L1	4.053	4.544	5.961	6.922
NKTR	4.189	5.027	4.912	4.785
NME1	1.568	2.975	6.079	6.589
NME2	4.150	5.880	9.252	9.184
NOL8	2.008	3.116	4.149	3.345
NOLC1	2.150	2.603	3.644	4.088
NOP10	1.844	4.299	7.288	6.829
NOP56	1.834	2.979	4.444	4.515
NPC2	4.593	4.673	7.247	8.708
NR3C1	2.315	3.362	4.524	3.882
NRD1	2.925	3.938	4.722	4.579
NSA2	1.993	2.909	4.738	3.923
NSRP1	1.807	2.913	3.599	3.763
NUCB2	6.055	5.577	6.164	9.922
NUP214	2.138	2.588	3.443	3.208
OAZ1	3.971	6.465	8.549	8.322
OCIAD1	1.861	2.601	3.352	4.610
ORMDL1	1.914	2.734	4.444	5.985
OS9	2.503	2.521	3.748	5.601
OSTC	4.401	5.122	5.979	10.045
OXR1	2.737	4.302	4.930	3.844
PABPC4	3.656	4.468	6.150	6.737

PAIP2	1.939	3.836	5.468	4.965
PALM2-AKAP2	1.413	2.791	3.808	3.483
PAPOLA	1.648	3.222	3.075	4.177
PARK7	2.393	3.448	5.200	6.901
PARP1	1.593	3.766	4.053	5.961
PCBP1	1.569	2.313	3.811	5.107
PCBP2	3.367	3.675	5.272	4.549
PCF11	2.480	3.549	4.890	3.275
PCMTD1	2.562	4.336	4.927	4.758
PDCD4	3.847	4.620	5.332	2.584
PDCD5	1.040	2.675	5.069	5.231
PDE4B	4.344	3.737	4.846	2.967
PDIA3	3.199	4.885	5.100	7.534
PDIA4	2.988	3.013	4.277	8.435
PDIA6	6.097	6.041	6.961	10.484
PDK1	3.603	3.243	3.663	4.599
PDXDC1	2.548	2.537	3.384	3.495
PEBP1	3.183	3.469	4.801	7.213
PECAM1	2.031	2.345	4.869	7.422
PELI1	5.821	5.178	6.723	7.682
PFN1	1.854	2.832	4.182	7.168
PGM3	2.550	2.320	2.961	4.538
PHIP	2.510	2.582	3.732	3.546
PHPT1	2.338	4.599	5.494	9.088
PIAS1	2.307	4.125	5.161	4.200
PIGF	1.194	3.010	4.505	5.055
PJA2	2.816	3.556	4.052	4.959
PNN	2.882	3.797	5.100	4.110
PNRC1	1.981	2.845	3.505	3.595
POLR2I	1.142	2.473	4.412	5.302
POLR2J	1.491	2.206	4.437	4.405
POLR2K	1.836	2.490	3.681	5.210
POLR2L	3.882	5.694	8.523	7.897
POMP	2.092	3.352	4.449	4.745
PPA1	0.846	2.536	3.985	4.356
PPAPDC1B	3.914	4.468	6.541	8.597
PPIG	3.448	3.800	4.179	4.217
PPIP5K2	2.432	2.907	3.673	3.945
PPM1K	2.171	2.781	3.126	4.571
PPP1R12A	1.967	2.850	2.831	3.874
PPP1R15A	3.557	4.556	5.400	5.372
PPP1R2	1.792	3.021	3.887	4.294
PPP2R5C	2.476	2.771	3.774	3.034
PPP6R3	2.800	3.133	3.691	2.975
PRDM1	1.860	3.547	4.032	2.567
PRDM2	2.403	2.584	3.342	3.648
PRDX1	3.739	4.179	5.613	9.301
PRDX2	2.604	3.568	6.293	7.876
PRDX5	2.837	4.271	7.057	6.767
PRKD3	3.329	4.233	5.633	2.945
PRPF38B	3.472	3.682	4.348	4.586
PRPF40A	1.996	2.615	3.580	3.489
PRRC2C	4.126	4.428	4.827	5.753
PSAP	3.998	5.010	5.941	8.112
PSENEN	1.801	2.603	4.657	5.425
PSMA1	2.524	3.597	5.480	5.141
PSMA2	2.403	4.267	5.828	6.342
PSMA3	4.346	5.227	6.620	6.478
PSMA4	1.653	4.406	5.158	4.951
PSMA6	1.369	2.479	4.600	6.318
PSMA7	2.611	3.547	5.326	6.356
PSMB1	3.654	5.128	6.331	8.297
PSMB3	2.732	4.050	5.084	7.870
PSMB4	4.888	6.203	8.350	9.676
PSMB5	1.287	2.206	4.323	4.799
PSMB6	3.473	3.760	5.902	8.236
PSMB7	2.109	3.530	6.344	6.109
PSMB9	2.558	3.042	4.548	5.951

PSMC4	1.647	2.482	3.219	4.256
PSMC6	2.158	2.352	3.762	4.522
PSMD7	1.998	3.151	3.691	6.642
PSMD8	1.484	2.372	4.235	5.172
PSME2	3.335	7.112	8.670	7.342
PTGES3	2.356	3.608	4.999	4.335
PTP4A2	4.302	4.147	5.291	5.691
PTPN2	3.115	2.643	4.224	3.393
PTPN6	1.484	2.859	3.276	6.392
PUM2	2.652	2.914	4.290	4.005
QPCT	2.178	3.566	5.523	2.852
RAB1A	3.714	3.659	4.425	5.666
RAB2A	1.760	2.238	3.394	4.983
RAB30	4.424	3.340	5.500	2.861
RABAC1	3.225	4.113	5.930	6.428
RABGAP1L	1.737	3.363	3.801	3.189
RALGPS2	2.284	2.246	3.467	5.732
RAN	1.352	2.897	5.242	5.089
RANBP2	2.722	2.935	3.499	3.308
RAP1B	1.274	2.487	4.307	4.056
RAPGEF2	1.824	3.607	4.038	3.152
RASGRP3	2.589	2.953	2.502	5.831
RASSF6	0.992	2.346	3.699	4.795
RB1CC1	3.035	3.491	4.331	3.192
RBM25	5.048	5.870	6.348	5.683
RBM5	2.212	3.104	3.430	3.009
RBM6	5.888	5.716	7.123	4.914
RBM8A	1.849	3.290	4.666	6.416
RBX1	2.772	3.477	4.542	6.135
REEP5	3.999	5.575	7.118	7.687
REXO2	1.249	3.771	6.517	5.875
RGS1	5.269	3.846	3.407	5.713
RGS2	2.774	3.371	2.889	6.609
RHOA	2.726	4.126	5.584	6.138
RNASEK	3.908	5.618	7.071	9.147
RNASET2	1.997	2.968	5.162	2.554
RNMT	2.515	3.434	3.774	3.488
ROMO1	3.104	5.248	7.292	8.604
RPN1	3.737	4.940	5.585	7.830
RRBP1	3.306	3.871	4.958	4.933
RSF1	2.019	2.902	3.782	3.188
RSL1D1	1.946	3.217	5.033	4.869
RSL24D1	1.964	3.722	5.248	4.544
RSRC1	2.209	3.599	3.652	3.023
RSRC2	3.521	3.534	4.531	4.910
RWDD1	1.722	3.018	4.785	4.288
S100A11	2.977	2.031	4.203	6.022
S100A6	3.610	4.004	5.419	11.639
SAP18	2.409	3.340	4.440	4.064
SARS	2.860	3.405	3.594	5.612
SBDS	3.511	3.817	5.123	6.375
SCAPER	2.795	3.850	5.216	2.561
SCFD1	3.730	4.365	6.039	5.977
SDF2L1	1.625	2.411	3.242	6.789
SDHD	1.347	2.690	4.840	3.467
SEC13	2.198	2.122	4.153	5.553
SEC14L1	5.369	5.371	6.606	5.761
SEC24D	1.853	2.519	3.235	4.528
SEC31A	4.632	5.363	5.821	7.357
SEC61A1	2.978	3.985	6.602	7.024
SEC61G	3.978	6.238	8.328	9.013
SEC63	3.661	3.801	5.594	6.725
SECISBP2	1.799	2.835	4.411	3.352
SEL1L3	4.363	3.079	4.074	3.763
SELM	3.190	4.914	5.528	8.055
SELT	3.330	4.749	5.086	7.628
15-Sep	2.184	2.554	3.894	5.801
2-Sep	2.782	2.712	4.218	5.052

7-Sep	2.875	3.776	3.994	6.445
SEPW1	1.271	2.486	4.361	4.524
SERINC1	2.429	3.240	3.522	5.011
SERP1	1.645	3.049	4.525	5.556
SET	2.381	3.097	4.345	3.440
SF3B1	5.362	4.752	5.039	5.764
SF3B14	2.107	3.087	4.009	5.228
SF3B2	1.940	3.639	4.506	4.328
SF3B5	2.563	3.303	4.832	8.580
SFT2D1	1.420	2.837	3.799	4.430
SH3BGRL3	2.694	4.456	5.754	8.349
SH3GLB1	2.702	2.089	3.392	4.589
SHFM1	3.013	5.354	8.236	7.524
SIL1	2.002	2.267	3.708	4.361
SKP1	3.889	4.811	7.137	7.179
SLAMF1	0.717	3.102	4.838	6.285
SLAMF7	4.635	5.338	6.856	9.125
SLC25A3	2.452	3.052	4.879	4.860
SLC35B1	2.049	3.077	4.505	6.641
SLC38A2	2.261	3.161	3.143	4.404
SLC3A2	1.947	2.985	3.591	6.228
SLIRP	2.807	3.553	6.159	6.078
SLTM	3.155	3.911	5.053	4.527
SMCHD1	2.908	2.917	3.411	3.701
SND1	2.129	3.088	4.349	4.296
SNRPA1	1.894	3.426	4.940	3.093
SNRPC	1.752	2.384	4.490	5.189
SNRPD1	0.807	3.102	5.027	3.833
SNRPD2	3.373	6.426	9.119	8.482
SNRPE	1.181	2.632	5.447	6.897
SNRPG	3.217	4.017	6.887	6.054
SNRPN	3.201	3.961	5.964	6.915
SNURF	3.809	4.461	6.848	7.446
SNX29	3.189	2.618	3.470	3.284
SOD1	2.444	4.741	5.488	6.570
SP100	2.759	4.127	4.155	2.822
SPAG4	1.831	2.815	4.446	6.820
SPATS2	3.667	4.055	5.624	5.342
SQSTM1	2.930	3.016	3.520	3.618
SREK1	3.346	4.539	5.314	4.304
SRGN	4.484	5.771	6.938	10.270
SRI	0.950	2.380	4.190	4.706
SRP19	2.340	3.703	5.385	6.245
SRP54	3.290	3.722	4.390	4.972
SRP72	2.862	3.843	5.183	6.612
SRP9	2.258	2.870	4.529	5.619
SRPR	3.313	4.426	6.022	6.961
SRPRB	2.918	3.875	5.270	6.142
SRRM1	2.794	3.776	4.506	4.284
SRSF10	2.541	3.671	4.699	3.901
SRSF11	5.041	5.055	6.114	5.365
SRSF5	5.529	6.840	7.300	7.099
SRSF7	5.558	6.069	6.163	7.872
SSB	2.241	3.404	4.339	3.999
SSBP1	1.209	3.239	5.610	5.139
SSR1	2.816	4.050	4.445	7.364
SSR2	3.811	5.675	8.123	10.558
ST13	1.589	2.499	3.920	3.649
ST3GAL6	3.792	4.217	4.530	2.614
STAP1	3.251	3.049	4.517	0.881
STK17B	3.922	3.529	2.982	2.721
STK4	2.752	3.027	3.422	3.331
STRBP	2.141	3.337	3.380	3.266
STT3A	1.776	3.996	5.024	5.808
STT3B	2.640	3.792	4.606	5.039
SULF2	1.029	2.852	4.591	3.083
SUMO2	2.988	3.670	5.256	5.861
SVIP	3.889	6.000	7.448	6.703

SYF2	2.149	3.208	3.633	3.609
SYNCRIP	2.374	3.036	4.501	5.341
SYNJ2BP-COX16	2.036	2.917	3.990	4.187
TAF1D	2.827	3.244	5.628	2.891
TAF7	2.954	4.696	6.161	5.917
TAF9	2.120	3.094	4.713	4.476
TAX1BP1	3.992	5.390	6.429	6.195
TBCA	2.512	4.698	7.043	6.237
TCEA1	1.925	3.143	4.205	4.503
TCEB2	4.131	5.765	7.490	8.091
TCERG1	2.732	3.644	4.353	3.405
TCF4	1.695	2.964	5.326	4.928
TECR	2.979	3.355	6.147	6.394
TES	1.520	2.627	2.814	5.001
THRAP3	2.447	2.654	4.289	4.136
TM9SF2	3.158	3.798	5.028	6.031
TMBIM4	3.217	5.989	7.584	8.423
TMCO1	2.708	5.262	6.632	9.127
TMED10	2.984	4.188	5.890	8.207
TMED2	1.710	2.924	3.493	7.576
TMED4	1.845	3.464	5.277	5.240
TMED5	4.088	4.189	4.851	7.202
TMEM147	0.713	2.295	5.463	4.920
TMEM167A	2.282	2.928	4.917	5.062
TMEM179B	2.706	3.384	5.004	5.035
TMEM205	2.718	3.828	5.680	7.887
TMEM208	1.405	2.379	4.329	7.629
TMEM39A	3.212	3.993	4.848	5.678
TMEM59	5.884	6.312	7.965	10.154
TMEM66	3.137	3.675	4.043	6.874
TMEM85	1.024	3.792	5.795	6.213
TMF1	2.400	2.816	3.552	3.436
TNFRSF17	4.074	7.807	8.507	10.814
TOB1	2.534	2.920	2.760	6.042
TOMM5	1.778	3.639	6.249	6.073
TOMM6	2.295	3.899	5.480	6.320
TOMM7	3.217	6.143	8.741	7.685
TOP1	3.315	3.912	4.134	4.564
TPD52	2.958	3.309	3.914	5.500
TPI1	2.227	3.523	5.577	6.981
TPM4	2.883	2.957	4.667	3.768
TPR	2.471	3.130	4.100	4.162
TRA2A	6.669	6.937	7.869	6.426
TRA2B	4.938	5.720	5.847	5.302
TRIB1	3.184	3.077	4.256	5.202
TRMT112	3.913	5.938	8.307	8.395
TSTD1	2.338	3.348	5.083	5.701
TTC1	1.566	2.911	3.499	4.686
TTC17	2.281	3.365	4.949	3.325
TTC3	3.936	4.517	5.103	5.900
TUBA1B	2.848	3.092	4.247	4.865
TXN	3.636	7.107	9.439	8.777
TXNDC11	2.867	4.130	5.112	6.803
TXNDC15	1.315	2.821	4.147	5.660
U2SURP	3.044	4.439	4.754	4.633
UAP1	2.677	3.421	4.821	6.389
UBE2B	5.112	5.973	7.295	5.998
UBE2D3	2.134	3.161	3.841	5.568
UBE2J1	3.742	5.727	7.511	7.784
UBE2V1	1.065	2.625	4.123	4.555
UBL5	3.437	6.617	8.766	7.554
UCP2	1.345	2.301	4.693	5.988
UFC1	3.140	2.889	4.641	8.249
UFD1L	1.576	2.666	3.703	4.534
UFM1	4.503	4.404	5.546	5.915
UQCR10	1.887	3.294	5.705	7.013
UQCR11	3.744	6.818	9.635	8.980
UQCRB	3.214	5.300	7.879	7.252

UQCRH	2.902	4.201	7.702	7.077
UQCRHL	1.269	2.394	4.336	3.688
UQCRQ	3.990	6.708	8.960	9.198
URGCP-MRPS24	1.746	3.009	6.447	6.923
USMG5	2.992	5.906	7.898	8.608
USO1	2.319	3.651	5.114	5.380
USP15	2.879	3.659	3.743	3.710
USP16	3.150	4.033	4.257	4.487
USP3	2.753	3.548	4.783	3.873
USP34	2.444	2.705	3.385	3.005
USP48	2.466	3.004	3.912	4.079
UTRN	3.236	3.644	3.629	3.617
VCP	1.453	3.312	3.514	6.216
VDAC2	2.153	3.751	4.814	4.742
VIM	5.307	5.826	7.074	9.984
VMP1	3.281	3.229	3.359	4.470
VPS29	1.700	2.834	4.129	5.582
WAC	2.233	3.154	4.211	3.739
WARS	1.468	2.901	1.881	5.783
WBSCR22	1.291	3.084	4.695	6.406
WDR43	1.800	2.672	4.303	2.649
WDR74	3.362	2.670	4.109	3.167
WDR83OS	1.631	4.937	7.106	6.324
WIPI1	1.621	3.461	4.528	5.456
WSB1	2.758	3.078	3.588	4.237
XRCC5	1.790	4.143	4.912	5.049
XRCC6	3.177	2.838	3.359	4.097
XRN1	2.502	3.733	3.670	3.580
YBX1	1.677	2.688	4.629	6.029
YME1L1	2.140	2.950	3.324	4.238
YPEL5	7.305	6.203	7.907	9.307
YTHDC1	2.981	3.929	3.868	3.266
YWHAB	2.354	3.955	3.393	5.116
YWHAE	2.467	3.820	5.255	5.290
YWHAZ	4.577	5.439	6.493	7.941
ZBTB8OS	1.679	2.265	3.637	4.739
ZC3H11A	3.090	3.885	4.621	5.000
ZC3H15	1.657	3.243	4.242	4.216
ZCCHC11	3.243	3.111	3.939	2.954
ZCCHC6	2.243	3.254	3.774	3.709
ZCCHC7	1.637	2.998	3.404	3.618
ZFAND6	1.579	3.058	4.196	3.246
ZFP36	2.524	3.581	3.877	4.915
ZNF207	2.616	3.107	5.728	4.425
ZNF638	1.739	2.779	3.385	3.324
ZNF706	4.053	6.291	9.134	9.395
