# Supplementary material for: Molecular signatures of multiple myeloma progression through single cell RNA-Seq
Source: Blood Cancer J. 2019 Jan 3;9(1):2. doi: 10.1038/s41408-018-0160-x (PMC6318319; doi:10.1038/s41408-018-0160-x)
Supplement: Supplementary file 2 — Supplemental Table S2 [file 41408_2018_160_MOESM2_ESM.pdf]

Supplemental Table S2. Significantly enriched Top 10 gene sets of each comparison.

L2 Vs. L1

Gene Set Name*	# of Genes in Overlap	P-value
OXIDATIVE_PHOSPHORYLATION	42	1.34 e-48
MYC_TARGETS_V1	28	1.8 e-27
MTORC1_SIGNALING	18	1.2 e-14
INTERFERON_GAMMA_RESPONSE	12	3.33 e-8
ANDROGEN_RESPONSE	9	6.05 e-8
UNFOLDED_PROTEIN_RESPONSE	9	1.6 e-7
ADIPOGENESIS	11	2.99 e-7
PROTEIN_SECRETION	7	7.03 e-6
INTERFERON_ALPHA_RESPONSE	7	7.52 e-6
APOPTOSIS	8	2.62 e-5

L3 Vs. L1

Gene Set Name*	# of Genes in Overlap	P-value
MYC_TARGETS_V1	61	2.64 e-63
OXIDATIVE_PHOSPHORYLATION	59	2.86 e-60
MTORC1_SIGNALING	35	1.26 e-27
UNFOLDED_PROTEIN_RESPONSE	20	1.85 e-16
PROTEIN_SECRETION	15	7.19 e-12
ADIPOGENESIS	20	1.2 e-11
DNA_REPAIR	17	5.72 e-11
INTERFERON_GAMMA_RESPONSE	19	9.51 e-11
ANDROGEN_RESPONSE	13	2.14 e-9
REACTIVE_OXIGEN_SPECIES_PATHWAY	9	2.7 e-8

L4 Vs. L1

Gene Set Name*	# of Genes in Overlap	P-value
MYC_TARGETS_V1	59	9.44 e-62
OXIDATIVE_PHOSPHORYLATION	58	3.18 e-60
MTORC1_SIGNALING	36	1.09 e-29
UNFOLDED_PROTEIN_RESPONSE	19	1.01 e-15
PROTEIN_SECRETION	17	1.33 e-14
ANDROGEN_RESPONSE	15	6.89 e-12
ADIPOGENESIS	19	3.64 e-11
DNA_REPAIR	16	2.29 e-10
HYPOXIA	18	2.9 e-10
INTERFERON_GAMMA_RESPONSE	18	2.9 e-10

L3 Vs L2

Gene Set Name*	# of Genes in Overlap	P-value
OXIDATIVE_PHOSPHORYLATION	58	2.3 e-65
MYC_TARGETS_V1	46	8.77 e-47
MTORC1_SIGNALING	25	4.28 e-19
UNFOLDED_PROTEIN_RESPONSE	15	2.2 e-12
ADIPOGENESIS	17	1.07 e-10
DNA_REPAIR	13	1.33 e-8
INTERFERON_GAMMA_RESPONSE	14	5.61 e-8
REACTIVE_OXIGEN_SPECIES_PATH\	8	6.02 e-8
GLYCOLYSIS	13	3.92 e-7
PROTEIN_SECRETION	8	1.13 e-5

L4 Vs L3

Gene Set Name*	# of Genes in Overlap	P-value
MTORC1_SIGNALING	16	9.52 e-14
PROTEIN_SECRETION	12	7.54 e-13
MYC_TARGETS_V1	12	2.43 e-9
OXIDATIVE_PHOSPHORYLATION	12	2.43 e-9
ANDROGEN_RESPONSE	9	1.15 e-8
HYPOXIA	11	2.34 e-8
UNFOLDED_PROTEIN_RESPONSE	8	4.34 e-7
GLYCOLYSIS	9	2.74 e-6
ADIPOGENESIS	8	2.58 e-5
FATTY_ACID_METABOLISM	7	4.49 e-5

* Hallmark gene set database
