# Supplementary material for: Molecular signatures of multiple myeloma progression through single cell RNA-Seq
Source: Blood Cancer J. 2019 Jan 3;9(1):2. doi: 10.1038/s41408-018-0160-x (PMC6318319; doi:10.1038/s41408-018-0160-x)
Supplement: Supplementary file 3 — Supplemental Table S3 [file 41408_2018_160_MOESM3_ESM.pdf]

Supplemental Table S3. Differentially expressed proteasome subunit gene list among the groups.

GeneID	Fold Change_ L2 Vs. L1	L2 vs L1.pValue	Fold Change_ L3 Vs. L1	L3 vs L1.pValue	Fold Change_ L4 Vs. L1	L4 vs L1.pValue	Fold Change_ L3 Vs. L2	L3 vs L2.pValue	Fold Change_ L4 Vs. L2	L4 vs L2.pValue	Fold Change_ L4 Vs. L3	L4 vs L3.pValue
PSMA1	2.1	0.157499943	7.8	5.74E-07	6.1	5.50E-05	3.7	6.33E-05	2.9	0.006235539	0.8	0.909612673
PSMA2	3.6	0.001434682	10.7	3.10E-09	15.3	6.12E-10	3.0	0.001185904	4.2	5.84E-05	1.4	0.73506502
PSMA3	1.8	0.286410388	4.8	0.000117724	4.4	0.00103196	2.6	0.004013146	2.4	0.031818208	0.9	0.991600591
PSMA4	6.7	2.06E-07	11.4	8.15E-10	9.8	4.23E-08	1.7	0.25095363	1.5	0.624464518	0.9	0.974169067
PSMA6	2.2	0.08133215	9.4	1.47E-09	30.9	4.83E-10	4.3	3.23E-07	14.3	4.83E-10	3.3	0.001237525
PSMA7	1.9	0.149689303	6.6	8.45E-08	13.4	4.85E-10	3.4	8.74E-06	7.0	5.62E-10	2.0	0.088381658
PSMB1	2.8	0.012644392	6.4	1.64E-06	25.0	4.83E-10	2.3	0.014211523	9.0	5.00E-10	3.9	0.000266676
PSMB3	2.5	0.037833748	5.1	5.14E-05	35.2	4.83E-10	2.0	0.05427102	14.1	4.83E-10	6.9	1.04E-07
PSMB4	2.5	0.060561892	11.0	7.62E-09	27.6	4.83E-10	4.4	5.88E-06	11.1	4.95E-10	2.5	0.054182072
PSMB5	1.9	0.234733255	8.2	4.98E-08	11.4	3.46E-09	4.3	1.19E-06	6.0	6.87E-08	1.4	0.757274115
PSMB6	1.2	0.938468407	5.4	3.19E-05	27.2	4.83E-10	4.4	1.45E-06	22.3	4.83E-10	5.0	1.54E-05
PSMB7	2.7	0.014652931	18.8	4.83E-10	16.0	4.85E-10	7.0	4.93E-10	6.0	3.07E-08	0.8	0.958524517
PSMB9	1.4	0.752256074	4.0	0.000793605	10.5	1.09E-08	2.8	0.001125336	7.5	1.50E-09	2.6	0.019658583
PSMC4	1.8	0.155333807	3.0	0.001326378	6.1	8.63E-08	1.7	0.113498128	3.4	9.49E-06	2.1	0.043345426
PSMC6	1.1	0.972831466	3.0	0.004652515	5.1	2.72E-05	2.7	0.000890524	4.5	1.18E-06	1.7	0.31922884
PSMD7	2.2	0.034666972	3.2	0.001178226	25.0	4.83E-10	1.5	0.416110366	11.2	4.83E-10	7.7	5.23E-10
PSMD8	1.9	0.171902442	6.7	2.80E-08	12.9	4.85E-10	3.6	1.65E-06	7.0	5.20E-10	1.9	0.130317664
PSME2	13.7	4.83E-10	40.4	4.83E-10	16.1	4.83E-10	2.9	0.000244865	1.2	0.947161727	0.4	0.018295548
