# Supplementary material for: Molecular signatures of multiple myeloma progression through single cell RNA-Seq
Source: Blood Cancer J. 2019 Jan 3;9(1):2. doi: 10.1038/s41408-018-0160-x (PMC6318319; doi:10.1038/s41408-018-0160-x)
Supplement: Supplementary file 4 — Supplemental Table S4 [file 41408_2018_160_MOESM4_ESM.pdf]

Supplemental Table S4. 311 genes list of most significantly expressed by disease progression (FC≥2 & p<0.05).

GeneID	Fold change_L2 Vs. L1	L2 vs L1.pValue	Fold change_L3 Vs. L1	L3 vs L1.pValue	Fold change_L4 Vs. L1	L4 vs L1.pValue
ACTG1	2.9	0.019374551	6.9	4.84E-06	5.8	0.000147028
AIG1	2.5	0.01513755	7.4	5.28E-09	52.8	4.83E-10
AKAP2	2.7	0.000977634	5.7	7.08E-09	4.5	4.26E-06
ANAPC16	2.5	0.040324656	6.0	6.32E-06	23.3	4.83E-10
ANP32B	3.2	0.000378023	11.1	4.83E-10	2.5	0.026614235
ANXA2	4.6	6.16E-05	14.3	4.91E-10	49.4	4.83E-10
APRT	3.8	0.000407542	6.5	1.02E-06	16.1	4.87E-10
ARF1	3.9	0.00060403	11.4	1.00E-09	43.5	4.83E-10
ARID4B	2.6	0.001475232	2.4	0.008238957	2.6	0.006406599
ARPC2	3.0	0.004339577	6.6	3.27E-07	7.6	2.39E-07
ARPC3	3.5	0.001399809	13.0	5.10E-10	26.5	4.83E-10
ATG3	3.1	0.003885021	9.1	3.64E-09	6.6	3.63E-06
ATP5A1	4.2	3.10E-05	15.7	4.83E-10	11.1	7.21E-10
ATP5B	4.8	8.66E-06	14.6	4.83E-10	21.6	4.83E-10
ATP5G1	4.0	0.00017472	12.4	4.99E-10	83.6	4.83E-10
ATP5G2	6.7	9.13E-10	29.0	4.83E-10	21.5	4.83E-10
ATP5H	2.9	0.00286549	9.6	5.90E-10	10.3	1.02E-09
ATP5J	2.5	0.029773862	9.9	1.04E-09	23.9	4.83E-10
ATP5J2	5.0	5.52E-07	32.1	4.83E-10	24.9	4.83E-10
ATP5J2-PTCD1	2.7	2.32E-05	8.7	4.83E-10	7.3	4.83E-10
ATP5O	3.7	0.00048663	21.4	4.83E-10	29.7	4.83E-10
ATP6V0E1	3.9	0.00237472	15.8	6.26E-10	26.3	4.84E-10
BANF1	6.0	5.67E-07	32.2	4.83E-10	69.9	4.83E-10
BSCL2	2.3	0.033880416	19.8	4.83E-10	43.6	4.83E-10
BST2	4.3	0.000349783	14.3	5.41E-10	3.7	0.009009121
BTF3	4.3	1.59E-05	14.7	4.83E-10	15.9	4.83E-10
BTG2	3.0	0.00766454	8.1	1.24E-07	19.1	4.84E-10
BUD31	2.6	0.01988751	8.0	2.89E-08	5.7	2.54E-05
C11orf31	4.2	3.11E-05	13.3	4.83E-10	9.2	6.41E-09
C11orf58	5.3	4.64E-07	14.8	4.83E-10	7.5	6.63E-08
C12orf57	2.3	0.043568437	7.4	2.58E-08	39.8	4.83E-10
C14orf2	5.5	1.32E-06	29.3	4.83E-10	28.7	4.83E-10
C15orf63	3.5	0.000267459	21.0	4.83E-10	7.8	5.04E-08
C19orf10	2.5	0.016735876	13.4	4.83E-10	22.8	4.83E-10
C19orf53	3.0	0.007114052	12.7	5.05E-10	7.8	6.95E-07
C19orf70	3.3	0.000406069	18.2	4.83E-10	13.4	4.83E-10
C19orf79	5.9	1.21E-08	35.6	4.83E-10	26.5	4.83E-10
C6orf48	2.5	0.026615415	19.3	4.83E-10	2.7	0.033695495
C7orf23	3.9	0.001807744	9.7	1.20E-07	5.9	0.000212091
C8orf59	2.6	0.009969754	8.9	9.63E-10	9.7	2.02E-09
CADPS2	2.2	0.025191221	3.1	0.001007075	2.3	0.043850888
CALM2	4.7	1.91E-05	13.5	4.86E-10	31.4	4.83E-10
CALU	2.5	0.040993599	7.0	9.97E-07	11.7	4.99E-09
CANX	4.7	6.82E-08	14.7	4.83E-10	10.8	4.83E-10
CAST	4.4	2.16E-06	8.7	5.04E-10	6.1	2.44E-07
CBWD1	3.3	1.91E-06	10.5	4.83E-10	9.9	4.83E-10
CBWD2	2.6	0.000301155	6.8	4.83E-10	8.6	4.83E-10
CCDC144A	3.7	6.85E-08	4.8	1.63E-09	3.4	1.86E-05
CCNDBP1	3.1	0.008847033	12.0	1.41E-09	4.2	0.002618116
CCNI	2.8	0.005738446	5.9	6.84E-07	7.3	1.90E-07
CCT8	3.6	0.000523016	11.3	5.45E-10	3.8	0.002434448
CD46	2.3	0.037578262	5.1	5.65E-06	15.4	4.83E-10
CD48	5.1	6.63E-05	24.2	4.83E-10	57.4	4.83E-10
CD53	3.6	0.001887523	14.2	5.32E-10	42.6	4.83E-10
CDK2AP2	2.7	0.019409846	5.8	1.12E-05	66.8	4.83E-10
CFLAR	2.3	0.023895872	2.5	0.023167295	35.8	4.83E-10
CHCHD2	3.2	0.002026664	15.6	4.83E-10	39.2	4.83E-10
CHMP2A	2.7	0.01545408	11.3	5.74E-10	10.1	1.02E-08
CIRBP	3.1	0.001681257	8.7	9.64E-10	2.5	0.042330091
CKLF	2.7	0.008146445	4.0	0.000189132	13.4	4.87E-10
CKLF-CMTM1	2.3	0.021341099	3.3	0.000846909	10.7	5.20E-10
CLIC1	4.2	0.000217159	14.8	4.88E-10	72.2	4.83E-10
CLPTM1L	3.0	0.000567916	6.3	7.49E-09	21.3	4.83E-10
CNBP	3.3	0.009985483	6.6	2.86E-05	12.1	1.23E-07
COPB1	2.2	0.040282232	6.1	6.72E-08	4.6	3.33E-05
COPB2	2.7	0.006296468	4.0	0.0001521	6.3	1.27E-06
COX17	6.0	1.83E-07	13.4	4.83E-10	26.5	4.83E-10
COX20	2.6	0.010198525	8.6	1.17E-09	8.6	1.00E-08
COX5A	4.7	2.37E-06	26.2	4.83E-10	9.2	1.99E-09
COX5B	7.2	2.41E-09	38.8	4.83E-10	26.8	4.83E-10
COX6A1	4.5	2.54E-05	14.0	4.83E-10	30.4	4.83E-10
COX6B1	7.2	2.01E-09	47.0	4.83E-10	42.1	4.83E-10
COX6C	5.3	3.60E-07	29.2	4.83E-10	42.7	4.83E-10
COX7A2	4.2	1.30E-05	16.9	4.83E-10	28.7	4.83E-10
COX8A	4.9	1.45E-05	36.9	4.83E-10	53.9	4.83E-10
CREB3L2	5.0	6.77E-08	14.2	4.83E-10	10.9	4.84E-10
CSDE1	2.8	0.004681726	6.0	3.32E-07	9.3	2.02E-09
CSNK2B	2.4	0.043300884	6.0	4.21E-06	11.4	2.97E-09

CUTA	3.7	5.70E-05	14.6	4.83E-10	15.4	4.83E-10
CWC15	2.2	0.041858222	6.2	2.00E-07	3.1	0.007496096
DAP3	3.5	0.000254143	7.2	1.34E-08	32.6	4.83E-10
DBI	2.8	0.005696173	11.7	4.88E-10	36.9	4.83E-10
DDX18	2.6	0.000663844	4.2	4.05E-07	4.1	4.56E-06
DDX21	2.0	0.042898377	4.4	2.42E-06	2.8	0.005830984
DHRS7	2.5	0.030608448	7.8	3.46E-08	14.2	5.01E-10
DMTF1	2.5	0.01280265	3.9	0.000199567	2.9	0.013108261
DNAJB11	5.6	3.81E-07	6.6	1.79E-07	27.7	4.83E-10
DNAJC1	2.1	0.014911409	3.3	8.12E-05	3.0	0.001115623
DNAJC10	2.7	0.003185729	9.6	4.84E-10	33.1	4.83E-10
DNAJC3	3.0	0.000808651	2.9	0.002330247	20.6	4.83E-10
DST	2.8	2.30E-05	3.0	2.03E-05	7.5	4.83E-10
DYNLL1	7.1	1.86E-06	19.0	4.89E-10	39.1	4.83E-10
DYNLRB1	3.1	0.002984627	6.6	6.51E-07	30.6	4.83E-10
EAF2	5.5	2.08E-06	5.5	1.01E-05	21.2	4.83E-10
EDF1	2.2	0.048342922	11.0	4.87E-10	12.4	4.91E-10
EID1	3.4	0.00165478	8.9	8.95E-09	7.7	6.92E-07
EIF2A	2.3	0.039499468	10.0	7.15E-10	4.8	0.000106675
EIF2S2	2.1	0.020303078	4.4	4.05E-07	10.0	4.83E-10
EIF3E	8.2	6.53E-10	35.0	4.83E-10	34.9	4.83E-10
EIF3J	5.4	6.40E-09	10.6	4.83E-10	2.9	0.003109675
EIF3K	5.3	2.96E-06	21.4	4.83E-10	8.3	1.13E-07
EIF3L	2.4	0.029027922	12.1	4.90E-10	5.1	6.63E-05
EIF5B	1.9	0.020637731	3.4	2.91E-06	3.6	3.70E-06
ELL2	3.5	2.36E-05	5.2	8.89E-08	3.1	0.001407607
EPRS	2.9	0.00021373	4.9	6.90E-08	7.5	5.76E-10
ERLEC1	2.5	0.003910634	6.9	6.59E-10	32.9	4.83E-10
EVI2A	2.5	0.041766503	5.3	3.56E-05	13.7	7.14E-10
EVI2B	3.7	0.000262617	10.3	5.91E-10	11.1	9.83E-10
FCRL5	2.2	0.035543241	5.7	1.33E-07	13.9	4.83E-10
FNBP4	2.3	0.007239185	4.4	3.92E-07	2.4	0.014398004
FXYD5	4.8	1.85E-05	18.8	4.83E-10	24.5	4.83E-10
GAPDH	3.3	0.002043207	14.5	4.84E-10	38.8	4.83E-10
GARS	3.0	0.002281139	6.2	1.78E-07	5.1	1.94E-05
GLCCI1	3.2	0.000803292	13.9	4.83E-10	5.0	2.00E-05
GLRX	5.6	1.94E-05	24.8	4.83E-10	48.5	4.83E-10
GNG5	2.7	0.029941835	6.7	4.72E-06	24.7	4.83E-10
GNG7	3.5	0.004789397	14.5	8.64E-10	12.8	2.95E-08
GNL3	3.5	0.000489862	6.8	8.90E-08	10.5	1.06E-09
GPRC5D	5.7	4.66E-07	12.6	4.85E-10	240.2	4.83E-10
GTF2A2	3.9	0.000542162	13.3	5.10E-10	13.0	1.25E-09
HIGD2A	4.3	0.000206993	25.7	4.83E-10	10.9	2.02E-08
HIST1H1C	3.9	0.000364202	12.4	5.13E-10	4.8	0.000290446
HIST1H2AC	3.7	0.002032857	7.8	1.12E-06	3.4	0.017180408
HIST1H2BK	3.8	0.001120411	8.2	2.88E-07	8.2	2.17E-06
HLA-A	2.9	0.001117668	6.2	1.99E-08	3.7	0.000346817
HLA-E	3.4	0.000778775	7.1	5.19E-08	5.8	7.52E-06
HLA-F	2.7	0.000478119	3.1	0.00025111	12.9	4.83E-10
HLA-G	2.3	7.09E-05	3.2	1.70E-07	3.5	1.45E-07
HMGN3	2.9	0.011477109	5.7	1.76E-05	4.8	0.000451192
HNRNPA1	3.7	0.000100667	13.6	4.83E-10	4.7	5.04E-05
HNRNPA1L2	2.7	0.001506618	8.1	4.88E-10	3.4	0.00037224
HNRNPC	3.5	0.001473315	8.6	2.36E-08	19.4	4.83E-10
HNRNPU	3.0	0.002813238	7.3	2.69E-08	6.5	1.34E-06
HSPA9	3.1	0.001419542	6.1	3.42E-07	3.8	0.000762592
HSPE1	3.3	0.003317777	14.4	4.89E-10	8.7	2.98E-07
ICAM3	3.4	3.53E-05	14.1	4.83E-10	4.4	1.24E-05
IER3IP1	2.6	0.020623018	9.3	4.35E-09	28.6	4.83E-10
IFITM2	11.5	7.91E-10	14.0	6.47E-10	5.5	0.000263084
IFNAR1	2.2	0.03887177	4.8	4.06E-06	12.8	4.83E-10
IK	2.4	0.022492473	6.1	2.26E-07	2.8	0.016085891
ISG20	5.0	1.13E-05	10.9	7.72E-10	10.8	5.28E-09
ITGA4	2.9	0.001168873	4.8	1.52E-06	4.7	1.23E-05
JTB	2.4	0.027291441	8.0	4.41E-09	26.8	4.83E-10
KDELR2	2.2	0.03380522	7.2	4.35E-09	15.6	4.83E-10
KIAA0368	2.1	0.009826778	2.3	0.004793128	3.0	0.000381442
LARS	3.5	1.34E-05	5.3	3.10E-08	6.0	2.90E-08
LDHB	2.7	0.019028004	28.0	4.83E-10	15.0	5.61E-10
LMAN1	2.3	0.017165046	3.4	0.000434824	13.9	4.83E-10
LSM5	3.2	0.001904432	11.3	4.98E-10	12.4	5.43E-10
LUC7L3	3.3	0.000158284	4.4	6.80E-06	3.8	0.000222089
MATR3	3.1	0.000994177	4.0	8.59E-05	3.1	0.005740556
MBD4	3.2	0.0003753	3.5	0.000297868	2.8	0.010786521
MESDC2	4.5	8.83E-07	19.0	4.83E-10	26.0	4.83E-10
MGST3	2.6	0.013443095	6.7	1.42E-07	57.4	4.83E-10
MIER1	2.1	0.034008384	2.9	0.001369938	4.1	3.11E-05
MRPL33	3.1	0.002536834	5.4	5.85E-06	9.9	4.23E-09
MRPL53	2.5	0.014724742	7.7	5.28E-09	8.4	1.11E-08
MRPS21	3.5	0.002668854	22.4	4.83E-10	12.1	8.74E-09
MRPS24	2.9	0.007919959	38.6	4.83E-10	46.3	4.83E-10
MTRNR2L2	2.9	0.000860538	9.8	4.83E-10	2.9	0.004819433

MYL12A	6.2	1.07E-06	8.5	5.94E-08	75.1	4.83E-10
NARS	2.8	0.001570273	5.5	6.91E-08	6.2	7.17E-08
NCL	2.3	0.012540733	4.8	5.33E-07	4.7	4.29E-06
NDUFA11	5.1	3.84E-08	23.0	4.83E-10	20.5	4.83E-10
NDUFA13	3.4	0.000365244	9.8	5.07E-10	12.8	4.86E-10
NDUFA2	3.1	0.002936958	20.2	4.83E-10	12.2	7.22E-10
NDUFA4	8.9	4.88E-10	53.5	4.83E-10	57.8	4.83E-10
NDUFA6	2.6	0.019684584	11.5	5.43E-10	16.9	4.84E-10
NDUFAF3	4.0	0.000152697	8.7	5.02E-09	24.9	4.83E-10
NDUFB1	6.7	5.33E-10	17.0	4.83E-10	16.9	4.83E-10
NDUFB10	3.0	0.002383081	4.5	4.61E-05	10.4	1.05E-09
NDUFB2	4.0	0.000154401	24.9	4.83E-10	13.6	5.16E-10
NDUFB3	2.5	0.035054742	11.8	6.85E-10	27.3	4.83E-10
NDUFB4	6.2	4.95E-08	13.9	4.83E-10	14.8	4.83E-10
NDUFB6	4.0	7.17E-05	19.9	4.83E-10	15.6	4.83E-10
NDUFB7	4.3	2.62E-05	18.8	4.83E-10	17.7	4.83E-10
NDUFB8	4.6	2.58E-05	29.8	4.83E-10	41.1	4.83E-10
NDUFB9	2.7	0.016620035	15.3	4.83E-10	12.8	6.46E-10
NDUFC1	2.6	0.036308823	9.9	8.05E-09	8.9	3.23E-07
NDUFC2	6.3	1.07E-07	22.7	4.83E-10	20.5	4.83E-10
NDUFC2-KCTD14	6.1	1.69E-07	21.3	4.83E-10	19.3	4.83E-10
NDUFS6	3.5	0.000653121	9.5	9.50E-10	4.2	0.00058328
NDUFS8	2.9	0.005861805	10.1	6.53E-10	9.3	1.14E-08
NDUFV2	2.7	0.024735762	8.8	8.00E-08	11.2	2.32E-08
NEDD8	4.4	0.000560257	22.3	4.83E-10	36.6	4.83E-10
NEDD8-MDP1	3.4	0.000655664	12.2	4.85E-10	24.3	4.83E-10
NFKBIA	2.7	0.006302336	3.2	0.002035064	10.3	8.64E-10
NGLY1	2.3	0.019393796	5.5	2.29E-07	4.9	9.27E-06
NHP2	3.7	0.000120256	13.1	4.83E-10	23.2	4.83E-10
NME1	2.7	0.023644967	22.8	4.83E-10	32.5	4.83E-10
NME2	3.3	0.000589189	34.3	4.83E-10	32.8	4.83E-10
NOL8	2.2	0.043975189	4.4	1.47E-05	2.5	0.028856903
NOP10	5.5	2.15E-05	43.5	4.83E-10	31.7	4.83E-10
NOP56	2.2	0.038438056	6.1	1.03E-07	6.4	3.51E-07
NSRP1	2.2	0.022361388	3.5	9.26E-05	3.9	6.18E-05
OAZ1	5.6	3.81E-08	23.9	4.83E-10	20.4	4.83E-10
PAIP2	3.7	0.001333486	11.5	1.80E-09	8.1	1.83E-06
PALM2-AKAP2	2.6	0.001247105	5.3	1.46E-08	4.2	7.32E-06
PAPOLA	3.0	0.000619221	2.7	0.005360359	5.8	3.48E-07
PARP1	4.5	1.50E-07	5.5	2.12E-08	20.6	4.83E-10
PCMTD1	3.4	0.000363669	5.2	3.66E-06	4.6	8.40E-05
PDCD5	3.1	0.001837616	16.3	4.83E-10	18.3	4.83E-10
PDIA3	3.2	0.000861672	3.7	0.000359981	20.2	4.83E-10
PHPT1	4.8	2.51E-05	8.9	1.35E-08	107.7	4.83E-10
PIAS1	3.5	0.000306148	7.2	2.10E-08	3.7	0.001240922
PIGF	3.5	0.000414582	9.9	6.00E-10	14.5	4.85E-10
POLR2I	2.5	0.019164146	9.6	7.35E-10	17.9	4.83E-10
POLR2L	3.5	0.000147495	25.0	4.83E-10	16.2	4.83E-10
POMP	2.4	0.040976043	5.1	2.41E-05	6.3	7.52E-06
PPA1	3.2	0.000990473	8.8	1.09E-09	11.4	5.71E-10
PPP1R2	2.3	0.040574842	4.3	0.000148595	5.7	1.58E-05
PRDX5	2.7	0.003689516	18.6	4.83E-10	15.2	4.83E-10
PSMA2	3.6	0.001434682	10.7	3.10E-09	15.3	6.12E-10
PSMA4	6.7	2.06E-07	11.4	8.15E-10	9.8	4.23E-08
PSMB1	2.8	0.012644392	6.4	1.64E-06	25.0	4.83E-10
PSMB3	2.5	0.037833748	5.1	5.14E-05	35.2	4.83E-10
PSMB7	2.7	0.014652931	18.8	4.83E-10	16.0	4.85E-10
PSMD7	2.2	0.034666972	3.2	0.001178226	25.0	4.83E-10
PSME2	13.7	4.83E-10	40.4	4.83E-10	16.1	4.83E-10
PTPN6	2.6	0.007194657	3.5	0.000515337	30.0	4.83E-10
RABGAP1L	3.1	0.000460678	4.2	1.65E-05	2.7	0.010475724
RAN	2.9	0.011325773	14.8	4.89E-10	13.3	1.23E-09
RAP1B	2.3	0.045401627	8.2	1.02E-08	6.9	1.23E-06
RAPGEF2	3.4	7.09E-05	4.6	2.20E-06	2.5	0.02110744
RASSF6	2.6	0.015314996	6.5	1.89E-07	14.0	4.86E-10
RBM8A	2.7	0.013449875	7.0	2.45E-07	23.7	4.83E-10
REEP5	3.0	0.011864834	8.7	1.11E-07	12.9	3.84E-09
REXO2	5.7	1.10E-06	38.5	4.83E-10	24.7	4.83E-10
RHOA	2.6	0.04292391	7.3	3.59E-06	10.6	1.64E-07
RNASEK	3.3	0.000877879	9.0	9.89E-10	37.8	4.83E-10
ROMO1	4.4	1.03E-05	18.2	4.83E-10	45.3	4.83E-10
RPN1	2.3	0.041867924	3.6	0.000884431	17.1	4.83E-10
RSL1D1	2.4	0.017804531	8.5	7.76E-10	7.6	3.10E-08
RSL24D1	3.4	0.000879089	9.7	8.24E-10	6.0	7.17E-06
RWDD1	2.5	0.009874517	8.4	5.44E-10	5.9	4.72E-07
SDHD	2.5	0.033376253	11.3	8.75E-10	4.3	0.001007892
SEC61G	4.8	1.56E-06	20.4	4.83E-10	32.8	4.83E-10
SECISBP2	2.1	0.044380157	6.1	6.88E-09	2.9	0.003400805
SELM	3.3	0.002132931	5.1	3.86E-05	29.2	4.83E-10
SELT	2.7	0.045655169	3.4	0.013575521	19.7	5.46E-10
SERP1	2.6	0.010541612	7.4	2.34E-08	15.0	4.84E-10
SF3B2	3.2	5.03E-05	5.9	2.86E-09	5.2	2.75E-07

SFT2D1	2.7	0.015424056	5.2	1.84E-05	8.1	2.29E-07
SH3BGRL3	3.4	0.007348855	8.3	1.28E-06	50.4	4.83E-10
SHFM1	5.1	1.36E-05	37.4	4.83E-10	22.8	4.83E-10
SLAMF1	5.2	4.92E-06	17.4	4.83E-10	47.4	4.83E-10
SNRPD1	4.9	8.16E-06	18.6	4.83E-10	8.1	1.54E-07
SNRPD2	8.3	7.62E-10	53.6	4.83E-10	34.5	4.83E-10
SNRPE	2.7	0.011810322	19.2	4.83E-10	52.5	4.83E-10
SOD1	4.9	6.23E-05	8.3	2.96E-07	17.5	5.24E-10
SRGN	2.4	0.049539816	5.5	2.87E-05	55.2	4.83E-10
SRI	2.7	0.011284806	9.4	1.18E-09	13.5	5.02E-10
SRP19	2.6	0.02293224	8.3	2.30E-08	15.0	4.96E-10
SRPR	2.2	0.026510959	6.5	2.31E-09	12.5	4.83E-10
SRRM1	2.0	0.024675077	3.3	2.67E-05	2.8	0.001037505
SRSF5	2.5	0.015459424	3.4	0.000960648	3.0	0.009567031
SSB	2.2	0.038182628	4.3	4.24E-05	3.4	0.002357757
SSBP1	4.1	9.01E-05	21.1	4.83E-10	15.2	4.86E-10
SSR1	2.4	0.04944331	3.1	0.008014165	23.4	4.83E-10
SSR2	3.6	0.001237308	19.9	4.83E-10	107.4	4.83E-10
STT3A	4.7	1.03E-05	9.5	9.37E-10	16.4	4.83E-10
STT3B	2.2	0.03912477	3.9	0.000132578	5.3	8.38E-06
SULF2	3.5	5.85E-05	11.8	4.83E-10	4.2	7.36E-05
SVIP	4.3	1.48E-06	11.8	4.83E-10	7.0	1.17E-08
SYF2	2.1	0.044435617	2.8	0.003399639	2.8	0.008652797
TAF7	3.3	0.000924317	9.2	1.20E-09	7.8	1.38E-07
TAX1BP1	2.6	0.001262328	5.4	1.37E-08	4.6	2.26E-06
TBCA	4.5	1.47E-06	23.1	4.83E-10	13.2	4.83E-10
TCEA1	2.3	0.011608282	4.9	5.50E-07	6.0	1.02E-07
TCEB2	3.1	0.000261748	10.3	4.83E-10	15.6	4.83E-10
TCF4	2.4	0.020702074	12.4	4.83E-10	9.4	1.77E-09
TES	2.2	0.039633123	2.5	0.018980834	11.2	4.87E-10
TMBIM4	6.8	5.16E-09	20.6	4.83E-10	36.9	4.83E-10
TMCO1	5.9	7.67E-09	15.2	4.83E-10	85.6	4.83E-10
TMED4	3.1	0.004208803	10.8	7.19E-10	10.5	5.84E-09
TMEM147	3.0	0.001682252	26.9	4.83E-10	18.5	4.83E-10
TMEM85	6.8	7.88E-07	27.3	4.83E-10	36.5	4.83E-10
TNFRSF17	13.3	4.83E-10	21.6	4.83E-10	106.9	4.83E-10
TOMM5	3.6	0.00049183	22.2	4.83E-10	19.6	4.83E-10
TOMM6	3.0	0.011158894	9.1	8.21E-08	16.3	6.33E-10
TOMM7	7.6	2.74E-08	46.0	4.83E-10	22.1	4.83E-10
TRMT112	4.1	7.94E-05	21.0	4.83E-10	22.3	4.83E-10
TTC1	2.5	0.012464551	3.8	0.000250069	8.7	6.02E-09
TXN	11.1	4.83E-10	55.8	4.83E-10	35.3	4.83E-10
TXNDC11	2.4	0.007964491	4.7	8.27E-07	15.3	4.83E-10
TXNDC15	2.8	0.00411722	7.1	2.33E-08	20.3	4.83E-10
U2SURP	2.6	0.000873366	3.3	8.13E-05	3.0	0.00088317
UBE2J1	4.0	6.24E-05	13.6	4.83E-10	16.5	4.83E-10
UBE2V1	2.9	0.001731012	8.3	8.14E-10	11.2	5.00E-10
UBL5	9.1	4.88E-10	40.2	4.83E-10	17.4	4.83E-10
UQCR10	2.7	0.017609407	14.1	4.84E-10	34.9	4.83E-10
UQCR11	8.4	5.17E-10	59.3	4.83E-10	37.7	4.83E-10
UQCRB	4.2	4.34E-07	25.4	4.83E-10	16.4	4.83E-10
UQCRH	2.5	0.041368967	27.8	4.83E-10	18.1	4.85E-10
UQCRHL	2.2	0.009065742	8.4	4.83E-10	5.3	2.70E-08
UQCRQ	6.6	2.51E-09	31.3	4.83E-10	37.0	4.83E-10
URGCP-MRPS24	2.4	0.049726006	26.0	4.83E-10	36.2	4.83E-10
USMG5	7.5	2.92E-09	30.0	4.83E-10	49.0	4.83E-10
USO1	2.5	0.007330996	6.9	3.14E-09	8.3	1.53E-09
VCP	3.6	8.19E-05	4.2	3.71E-05	27.2	4.83E-10
VDAC2	3.0	0.007624343	6.3	4.20E-06	6.0	3.64E-05
WBSCR22	3.5	0.001208393	10.6	9.07E-10	34.7	4.83E-10
WDR83OS	9.9	1.48E-09	44.5	4.83E-10	25.9	4.83E-10
WIPI1	3.6	0.000650516	7.5	8.08E-08	14.3	5.03E-10
XRCC5	5.1	2.67E-07	8.7	5.63E-10	9.6	7.15E-10
YWHAE	2.6	0.002528292	6.9	6.15E-10	7.1	1.79E-09
ZC3H15	3.0	0.00089194	6.0	5.85E-08	5.9	6.19E-07
ZCCHC7	2.6	0.009103498	3.4	0.000770345	3.9	0.000365111
ZFAND6	2.8	0.009075086	6.1	1.33E-06	3.2	0.010168256
ZNF638	2.1	0.02200917	3.1	0.000139571	3.0	0.000794033
ZNF706	4.7	0.000154248	33.9	4.83E-10	40.6	4.83E-10
