# Supplementary material for: Molecular signatures of multiple myeloma progression through single cell RNA-Seq
Source: Blood Cancer J. 2019 Jan 3;9(1):2. doi: 10.1038/s41408-018-0160-x (PMC6318319; doi:10.1038/s41408-018-0160-x)
Supplement: Supplementary file 5 — Supplemental Table S5 [file 41408_2018_160_MOESM5_ESM.pdf]

Supplemental Table S5. 44 genes list of most consistently related to MM progression (FC≥2 & p<0.05).

Gene Symbol	Chromosome	Start-End	Description	GO Term	Fold Change L2 Vs. L1	L2 vs L1.pValue	Fold Change L3 Vs. L2	L3 vs L2.pValue	Fold Change L4 Vs. L3	L4 vs L3.pValue
AIG1	chr6	143382023-143661441	androgen-induced 1		2.5	0.01513755	3.0	6.45E-05	7.2	9.53E-10
ANAPC16	chr10	73975758-73995618	anaphase promoting complex subunit 16		2.5	0.040324656	2.4	0.009336875	3.9	0.000355666
ANXA2	chr15	60639350-60690185	annexin A2	UPR (ER stress)	4.6	6.16E-05	3.1	0.000494056	3.5	0.001695497
ARF1	chr1	228270361-228286913	ADP-ribosylation factor 1	UPR (ER stress)	3.9	0.00060403	3.0	0.000916458	3.8	0.000637969
ATP5G1	chr17	46970148-46973232	ATP synthase, H+ transporting, mitochondrial Fo complex, subunit C1 (subunit 9)	MITOCHONDRION	4.0	0.00017472	3.1	0.000251634	6.8	5.29E-08
ATP5J	chr21	27096791-27107965	ATP synthase, H+ transporting, mitochondrial Fo complex, subunit F6	MITOCHONDRION	2.5	0.029773862	4.0	1.95E-06	2.4	0.034583101
BSCL2	chr11	62457734-62477091	Berardinelli-Seip congenital lipodystrophy 2 (seipin)	ENDOPLASMIC RETICULUM	2.3	0.033880416	8.8	4.83E-10	2.2	0.040315923
C12orf57	chr12	7053203-7055165	chromosome 12 open reading frame 57		2.3	0.043568437	3.2	4.52E-05	5.4	5.51E-07
CD46	chr1	207925383-207968861	CD46 molecule, complement regulatory protein	UPR (ER stress)	2.3	0.037578262	2.2	0.009398674	3.0	0.001694595
CD53	chr1	111413821-111442558	CD53 molecule		3.6	0.001887523	3.9	2.62E-05	3.0	0.010965738
CDK2AP2	chr11	67273968-67276102	cyclin-dependent kinase 2 associated protein 2		2.7	0.019409846	2.1	0.037185656	11.6	4.90E-10
CHCHD2	chr7	56169266-56174187	coiled-coil-helix-coiled-coil-helix domain containing 2	UPR (ER stress), MITOCHONDRION	3.2	0.002026664	4.9	2.67E-08	2.5	0.02161909
CLIC1	chr6	31698358-31704341	chloride intracellular channel 1	MITOCHONDRION	4.2	0.000217159	3.5	7.09E-05	4.9	2.51E-05
CLPTM1L	chr5	1318000-1345002	CLPTM1-like		3.0	0.000567916	2.1	0.007079639	3.4	6.20E-05
DAP3	chr1	155658882-155708800	death associated protein 3	MITOCHONDRION	3.5	0.000254143	2.1	0.022693784	4.5	4.71E-06
DBI	chr2	120124504-120130122	diazepam binding inhibitor (GABA receptor modulator, acyl-CoA binding protein)	ENDOPLASMIC RETICULUM	2.8	0.005696173	4.1	4.93E-07	3.2	0.001534051
DNAJC10	chr2	183580999-183643255	DnaJ (Hsp40) homolog, subfamily C, member 10	ENDOPLASMIC RETICULUM	2.7	0.003185729	3.6	3.15E-07	3.5	5.78E-05
DYNLRB1	chr20	33104204-33128762	dynein, light chain, roadblock-type 1		3.1	0.002984627	2.1	0.033586917	4.7	1.59E-05
EIF2S2	chr20	32676115-32700085	eukaryotic translation initiation factor 2, subunit 2 beta, 38kDa		2.1	0.020303078	2.1	0.002374198	2.3	0.006824822
ERLEC1	chr2	54014068-54045956	endoplasmic reticulum lectin 1	UPR (ER stress),ENDOPLASMIC RETICULUM	2.5	0.003910634	2.8	2.74E-05	4.7	4.86E-08
EVI2A	chr17	29643428-29648767	ecotropic viral integration site 2A		2.5	0.041766503	2.1	0.036504855	2.6	0.024322319
FCRL5	chr1	157483167-157522310	Fc receptor-like 5		2.2	0.035543241	2.6	0.00034145	2.4	0.009654471
GAPDH	chr12	6643585-6647537	glyceraldehyde-3-phosphate dehydrogenase	UPR (ER stress), MITOCHONDRION	3.3	0.002043207	4.4	5.34E-07	2.7	0.015409877
GNG5	chr1	84964006-84972262	guanine nucleotide binding protein (G protein), gamma 5	MITOCHONDRION	2.7	0.029941835	2.5	0.011079611	3.7	0.001439582
GPRC5D	chr12	13093709-13103318	G protein-coupled receptor, family C, group 5, member D		5.7	4.66E-07	2.2	0.015066616	19.0	4.83E-10
IER3IP1	chr18	44681390-44702745	immediate early response 3 interacting protein 1	ENDOPLASMIC RETICULUM	2.6	0.020623018	3.5	2.98E-05	3.1	0.003842754
IFNAR1	chr21	34697214-34732128	interferon (alpha, beta and omega) receptor 1	UPR (ER stress)	2.2	0.03887177	2.2	0.006736728	2.7	0.004053715
JTB	chr1	153946745-153950451	jumping translocation breakpoint	MITOCHONDRION	2.4	0.027291441	3.4	1.76E-05	3.3	0.000601098
KDELR2	chr7	6500712-6523849	KDEL (Lys-Asp-Glu-Leu) endoplasmic reticulum protein retention receptor 2	ENDOPLASMIC RETICULUM	2.2	0.03380522	3.2	1.12E-05	2.2	0.039215467
MGST3	chr1	165600110-165625372	microsomal glutathione S-transferase 3	ENDOPLASMIC RETICULUM	2.6	0.013443095	2.6	0.001738765	8.6	5.60E-10
NDUFAF3	chr3	49057908-49060926	NADH dehydrogenase (ubiquinone) 1 alpha subcomplex, assembly factor 3	MITOCHONDRION	4.0	0.000152697	2.2	0.017782084	2.9	0.006592931
PSMB1	chr6	170844204-170862417	proteasome (prosome, macropain) subunit, beta type, 1	UPR (ER stress)	2.8	0.012644392	2.3	0.014211523	3.9	0.000266676
RBM8A	chr1	145507557-145513535	RNA binding motif protein 8A		2.7	0.013449875	2.6	0.002775395	3.4	0.001268406
RNASEK	chr17	6915736-6917852	ribonuclease, RNase K		3.3	0.000877879	2.7	0.000571602	4.2	2.21E-05
ROMO1	chr20	34287232-34288902	reactive oxygen species modulator 1	MITOCHONDRION	4.4	1.03E-05	4.1	2.25E-07	2.5	0.015674473
SH3BGRL3	chr1	26606213-26608013	SH3 domain binding glutamic acid-rich protein like 3		3.4	0.007348855	2.5	0.021858047	6.0	1.26E-05
SLAMF1	chr1	160579891-160617081	signaling lymphocytic activation molecule family member 1	UPR (ER stress)	5.2	4.92E-06	3.3	8.01E-05	2.7	0.012759535
SNRPE	chr1	203830740-203840280	small nuclear ribonucleoprotein polypeptide E		2.7	0.011810322	7.0	4.93E-10	2.7	0.010928505
SRGN	chr10	70847828-70864567	serglycin		2.4	0.049539816	2.2	0.02467871	10.1	7.07E-10
SSR2	chr1	155978839-155990758	signal sequence receptor, beta (translocon-associated protein beta)	ENDOPLASMIC RETICULUM	3.6	0.001237308	5.5	3.32E-08	5.4	7.11E-06
TMCO1	chr1	165693528-165738159	transmembrane and coiled-coil domains 1	ENDOPLASMIC RETICULUM	5.9	7.67E-09	2.6	0.000393168	5.6	1.25E-08
TXNDC15	chr5	134209460-134237323	thioredoxin domain containing 15		2.8	0.00411722	2.5	0.001851365	2.9	0.003492195
UQCR10	chr22	30163358-30166402	ubiquinol-cytochrome c reductase, complex III subunit X	MITOCHONDRION	2.7	0.017609407	5.3	1.11E-08	2.5	0.029829969
WBSCR22	chr7	73097898-73112551	Williams Beuren syndrome chromosome region 22		3.5	0.001208393	3.1	0.000328405	3.3	0.002018528
