# Supplementary figures and images for: Molecular signatures of multiple myeloma progression through single cell RNA-Seq
Source: Blood Cancer J. 2019 Jan 3;9(1):2. doi: 10.1038/s41408-018-0160-x (PMC6318319; doi:10.1038/s41408-018-0160-x)

Supplemental Figure S1

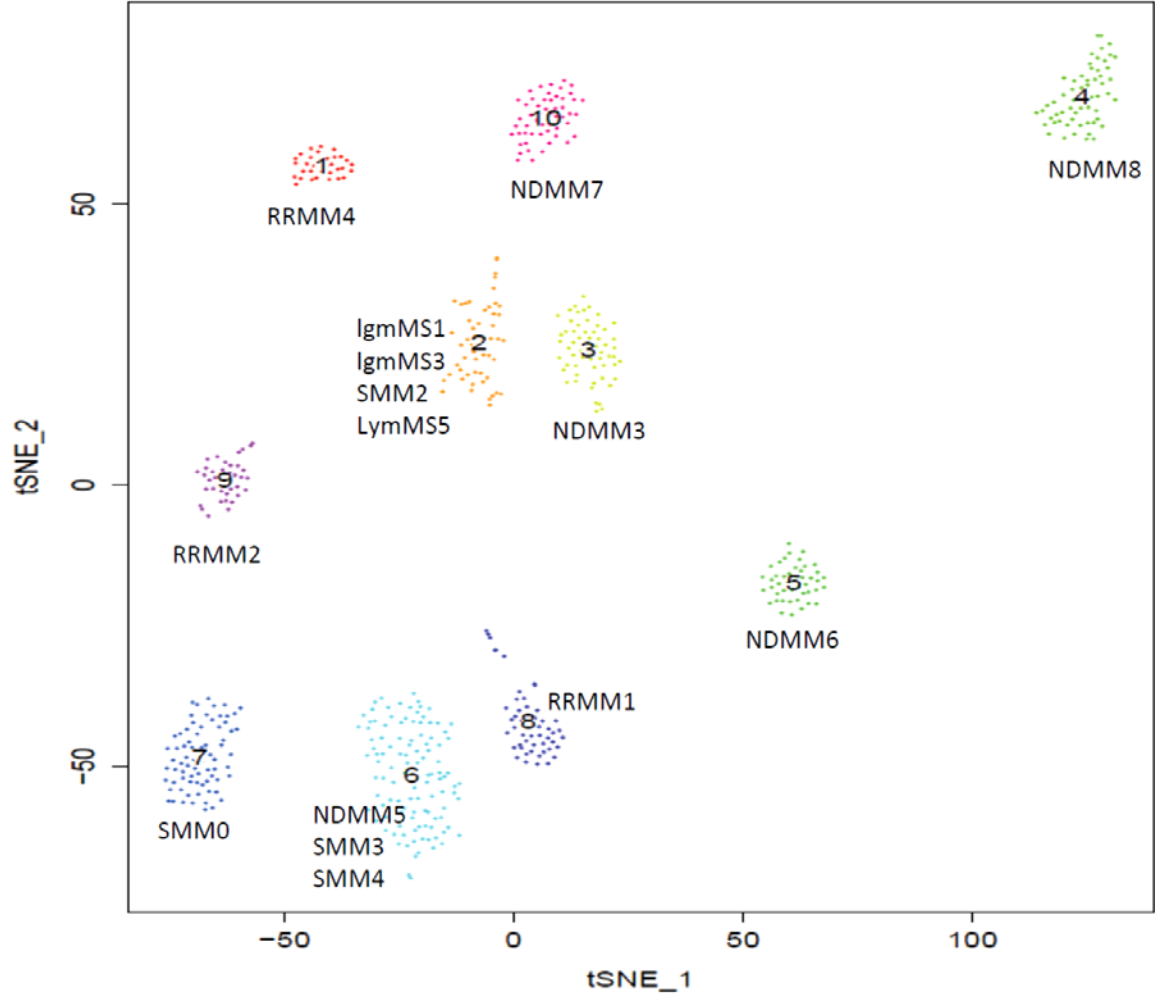

Supplement: Supplementary file 6 — Supplemental Figure S1 [file 41408_2018_160_MOESM6_ESM.pdf]

Supplemental Figure S2

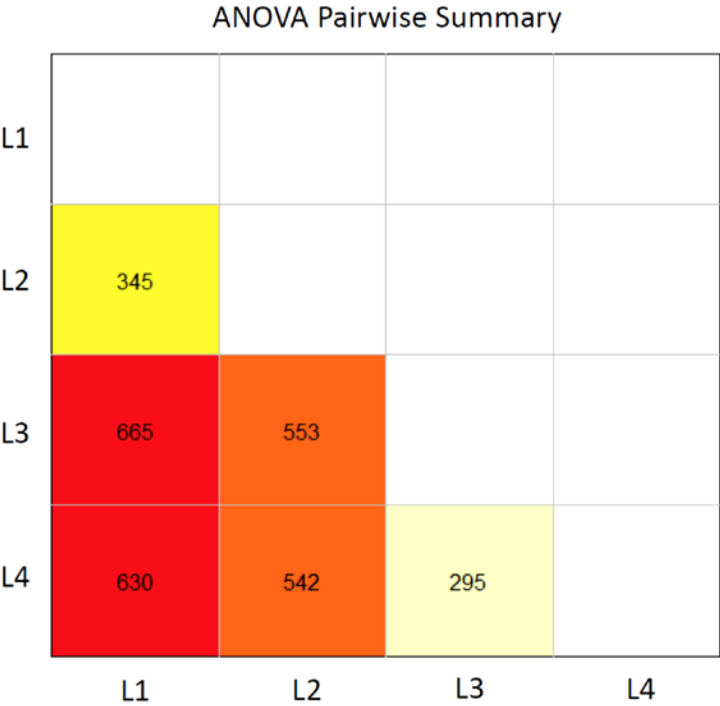

Supplement: Supplementary file 7 — Supplemental Figure S2 [file 41408_2018_160_MOESM7_ESM.pdf]
