# Supplementary material for: Molecular signatures of multiple myeloma progression through single cell RNA-Seq
Source: Blood Cancer J. 2019 Jan 3;9(1):2. doi: 10.1038/s41408-018-0160-x (PMC6318319; doi:10.1038/s41408-018-0160-x)
Supplement: Supplementary file 8 — Supplemental Figure Legends [file 41408_2018_160_MOESM8_ESM.docx]

**Supplemental Table Legends**

Supplemental Table S1. 790 genes list with moderately highly variable genes (log_2_(TPM+1).

Supplemental Table S2. Significantly enriched Top 10 gene sets of each comparison.

Supplemental Table S3. Differentially expressed proteasome subunit gene list among the groups.

Supplemental Table S4. 311 genes list of most significantly expressed by disease progression (FC≥2 & p<0.05).

Supplemental Table S5. 44 genes list of most consistently related to MM progression (FC≥2 & p<0.05).

**Supplemental Figure Legends**

Supplemental Figure S1. T-SNE plot of all 597 cells derived from 15 patients. t-SNE plot was generated by Seurat (version1.2) using genes were expressed in more than two cells with log_2_mean>1 and standard deviation (y)>1. Most of cells clustered mainly by individual patients reflecting the clonal genetic changes unique to each patient.

Supplemental Figure S2. ANOVA pairwise summary for 790 selected genes. ANOVA analysis was performed among the major groups of cells (log_2_ scale) and the significantly differentially expressed genes were selected using ANOVA *p*-value below 0.05. Each number of each comparison is the number of significantly expressed genes.
